# Do healthcare needs-based population segments predict outcomes among the elderly? Findings from a prospective cohort study in an urbanized low-income community

**DOI:** 10.1186/s12877-020-1480-9

**Published:** 2020-02-27

**Authors:** Jia Loon Chong, Lian Leng Low, David Bruce Matchar, Rahul Malhotra, Kheng Hock Lee, Julian Thumboo, Angelique Wei-Ming Chan

**Affiliations:** 1 Signature Program in Health Services and Systems Research, Duke-NUS Medical School, 8 College Road, Singapore, 169857 Singapore; 2 Department of Family Medicine and Continuing Care, Singapore General Hospital, 20 College Road, Singapore, 169856 Singapore; 3 SingHealth Duke-NUS Family Medicine Academic Clinical Program, Singapore, Singapore; 4 Department of Medicine (General Internal Medicine), Duke University Medical Center, Durham, NC USA; 5 Department of Internal Medicine, Singapore General Hospital, 20 College Road, Singapore, 169856 Singapore; 6 Centre for Ageing Research and Education, Duke-NUS Medical School, 8 College Road, Singapore, 169857 Singapore; 7 Department of Rheumatology and Immunology, Singapore General Hospital, 20 College Road, Singapore, 169856 Singapore

**Keywords:** Population segmentation, Community survey, Aging, Healthcare need, Health services research, Population health, Healthcare utilization, Decision-making, Consensus, Program evaluation

## Abstract

**Background:**

A rapidly ageing population with increasing prevalence of chronic disease presents policymakers the urgent task of tailoring healthcare services to optimally meet changing needs. While healthcare needs-based segmentation is a promising approach to efficiently assessing and responding to healthcare needs at the population level, it is not clear how available schemes perform in the context of community-based surveys administered by non-medically trained personnel. The aim of this prospective cohort, community setting study is to evaluate 4 segmentation schemes in terms of practicality and predictive validity for future health outcomes and service utilization.

**Methods:**

A cohort was identified from a cross-sectional health and social characteristics survey of Singapore public rental housing residents aged 60 years and above. Baseline survey data was used to assign individuals into segments as defined by 4 predefined population segmentation schemes developed in Singapore, Delaware, Lombardy and North-West London. From electronic data records, mortality, hospital admissions, emergency department visits, and specialist outpatient clinic visits were assessed for 180 days after baseline segment assignment and compared to segment membership for each segmentation scheme.

**Results:**

Of 1324 residents contacted, 928 agreed to participate in the survey (70% response). All subjects could be assigned an exclusive segment for each segmentation scheme. Individuals in more severe segments tended to have lower quality of life as assessed by the EQ-5D Index for health utility. All population segmentation schemes were observed to exhibit an ability to differentiate different levels of mortality and healthcare utilization.

**Conclusions:**

It is practical to assign individuals to healthcare needs-based population segments through community surveys by non-medically trained personnel. The resulting segments for all 4 schemes evaluated in this way have an ability to predict health outcomes and utilization over the medium term (180 days), with significant overlap for some segments. Healthcare needs-based segmentation schemes which are designed to guide action hold particular promise for promoting efficient allocation of services to meet the needs of salient population groups. Further evaluation is needed to determine if these schemes also predict responsiveness to interventions to meet needs implied by segment membership.

## Background

By 2050, the number of adults worldwide aged 60 years and over will for the first time in history match the number of individuals younger than 15 [1]. The world population is ageing, mainly due to rising life expectancy and decreasing fertility rates [2, 3]. Concurrent with this demographic shift is a rising proportion of individuals with chronic disease and more numerous and more complex healthcare needs. This presents healthcare policymakers with the challenge of properly understanding the changing nature of needs at a population level and developing health service interventions tailored to meet those needs [4].

A promising tool for solving this dual challenge is healthcare need-based population segmentation [5, 6]. As articulated by Steinbach [7] a healthcare service is needed if the recipient is capable of benefitting from the receipt of the service; this is manifest as reduced probability of transitioning into a worse health state. Segmentation based on needs is intended to assign the individual in the population into relatively homogenous and clinically meaningful groups, enabling efficient design of packages of health services designed to meet needs across the entire population [8]. Such tailoring is crucial as unmet needs increases risk of adverse outcomes [7, 9] while excess services leads to resource wastage [8].

Through a systematic review [10], we identified 3 needs-based segmentation schemes: from Delaware, Lombardy, and North-west London. The Delaware population segmentation scheme was designed in the context of one of the state’s healthcare transformation plans. The original Delaware scheme includes 4 main segments of the population namely the ‘elderly’, ‘adults’, ‘maternity and pediatrics’, as well as ‘special needs’ population groups. Each category of individuals has very different needs. For example, ‘adults’ may need multiple access channels for self-management while the ‘maternity and pediatrics’ population would need access to prenatal care as well as age-appropriate immunization coverage [11].

The Lombardy scheme was designed by researchers interested to assess ‘healthcare demand’ and cost borne by the Italian healthcare system of specific population segments using an Italian healthcare administrative database [12]. The population scheme utilized in the Lombardy study was adapted from the ‘Bridges to Health’ model of population segmentation [5].

The North West London segmentation scheme was created to divide its population into groups of people with similar needs so that models of care can be created and structured to address the needs of individual groups holistically, rather than being structured around different services and organisations [13]. For example, someone who is mostly healthy would require convenient access to routine medical care. Conversely, individuals who have multiple long-term conditions would likely need sustained continuity of care through longer primary care appointments and closely coordinated health services [14].

For understanding and planning for the health and health-related social needs of a population, an ideal segmentation tool should be practical, have demonstrable validity (e.g., ability to predict health outcomes and utilizations) and responsiveness (identifies individuals who are likely to benefit from interventions). Notably, the identified healthcare needs-based population segmentation schemes have been focused on individuals who utilize health services, as reflected by the predominance of electronic health record (EHR) variables in their segmentation inputs [15–17].

In the absence of an internationally available needs-based segmentation tool fulfilling these characteristics, we developed a segmentation scheme to be applied in both clinical and survey settings [18]. The tool is designed to capture individual features that relate to general medical needs (mapped to 6 Global Impression categories, based on the “Bridges to Health” framework) as well as 8 Complicating Factors that impact the risk and potential benefit of non-physician services; the latter were mapped to 3 Risk and Actionability categories (see Methods). A preliminary version for clinical use is the Singapore Simple Segmentation Tool (SST) and subsequently was implemented as a survey version (Additional file 1).

To the best of our knowledge, no prior study has investigated the predictive validity of healthcare needs-based segmentation schemes for clinically relevant outcomes when applied on a community survey dataset. Furthermore, it is not known if these healthcare needs-based segmentation schemes have predictive validity for healthcare utilization and health-related outcomes. In the context of a healthcare system moving more explicitly towards serving as stewards of population health [14] we sought to assess the practicality and predictive validity of 4 population segmentation schemes to promote this objective: Singapore [5], Delaware [11], Lombardy [12] and North-West London [13] using data from community-based survey of a low-income urbanized community in Singapore. We hypothesized that these segmentation schemes are able to distinguish groupings of our study population with distinct 180-day mortality and healthcare utilization.

## Methods

### Site

The baseline dataset for this prospective cohort study was obtained from a survey among residents of 12 public rental housing blocks in Singapore between December 2016 and March 2017. Residence in public rental housing is heavily government subsidized and is an indicator of low socio-economic status in Singapore [19] given that only financially needy households will qualify [20]. The community is contained within the SingHealth Regional Health System, one of three health clusters that serve as an organizing focus for population health services.

### Subjects

The Singapore Ministry of Health provided 1817 unique addresses of older adults aged 60 years and above living within the target community. Survey personnel then visited the addresses to determine the presence of eligible individuals and to conduct both the recruitment and survey. Eligible study subjects were Singaporean citizens or permanent residents aged 60 years and above who scored 7 or more on the Abbreviated Mental Test (AMT) or had a suitable proxy interviewee if they scored below 7 on the AMT. Respondents (either the study subject or their proxy) provided written consent.

### Measures

The survey was administered by non-healthcare trained surveyors in the subject’s home and captured health information (specific chronic disease, physical disability through adapted Barthel Index [21] and Lawton IADL scales [22], health promoting behaviours such as medication adherence and health knowledge, health utility score based on responses to the EQ5D Index [23, 24]) and social characteristics (social network, financial status, social stressors, etc) (see Additional file 2 for survey questionnaire). Patient self-reported data for clinical diagnoses and healthcare utilization have been demonstrated to have moderate to substantial degrees of concordance with administrative claims records [25, 26].

Health service utilization and mortality was assessed over 180 days after the baseline survey data. Data were obtained from the SingHealth electronic health record through the Health Intelligence System (eHints) [27]. eHints is a single enterprise data repository that integrates information from multiple healthcare transaction systems including administration, clinical and ancillary systems. Outcomes included mortality and healthcare utilization (hospital admissions, emergency department attendance, specialist outpatient clinic (SOC) attendances).

This study was approved by the SingHealth Centralized Institutional Review Board (CIRB2016/2242).

### Allocation of subjects to segments

Subjects were allocated to segments in each of the 4 schemes using a mapping algorithm that matches the original definition of specific population segments from the various alternative schemes considered (see Additional file 3). Study subjects were assigned into population segments by considering whether individual subjects’ characteristics met the pre-defined entry criteria for a specific segment and if not, they would be considered for the next segment based on an approximate highest to lowest healthcare need sequence. This ensures that subjects are assigned into population segments with the highest healthcare need for which they meet admission criteria.

As noted above, the Singapore scheme involved 6 Global Impression categories and a summary of Complicating Factors into 3 levels, denoted ‘Risk for adverse outcome and Actionability’ (RA) (Table 1). Combining 6 Global Impression Levels and 3 RA levels, the Singapore scheme comprises 18 health and social service need-based categories.

**Table 1:** RA levels definition based on SST complicating factor variables

Risk & actionability level	Definition
3: High Risk, high actionability	Any activity of daily living deficit or skilled nursing task need
2: Moderate risk, moderate actionability,	Not in level 3. Has any of the following deficit: Instrumental activity of daily living, social support, activation, disruptive behaviour
1: Low risk, low actionability	All others

### Statistical analysis

The study population was characterized using descriptive statistics on the various population segmentation schemes. Predictive validity of the various population segmentation schemes for the outcomes of mortality and healthcare utilization were plotted based on specific population segments’ mean outcome and confidence interval. Confidence intervals were computed based on normal standard errors for the outcome of SOC visit, Poisson standard errors for the outcome of ED visits and hospital admission, and binomial standard errors for the outcome of mortality. The average outcome rate for all subjects were plotted as a dashed line on the graph. Finally, in order to test the null hypothesis that the population segments have no relationship with the outcomes of mortality and healthcare utilizations, we perform Pearson Chi-squared tests between population segments and the following outcomes: any mortality, any hospital admission, any ED visit, and any SOC visit within the follow-up period of 180 days. Our alternative hypothesis is that population segments are related to outcomes of mortality and healthcare utilizations. We select a significance level of 0.05.

All analysis in this study was conducted using STATA (StataCorp. 2013. *Stata Statistical Software: Release 13*. College Station, TX: StataCorp LP). The report is consistent with the relevant items from the STROBE guidelines for reporting cohort studies [28] (see Additional file 4).

## Results

### Baseline

Of 1324 residents contacted, 928 subjects were recruited for this study (70% response rate). A proxy responded for 47 subjects (5.1%). Compared to the general Singapore population, individuals in this community tended to be older (median of 70 years old vs 41 years old for Singapore overall [29]) with low socioeconomic indicators (100% in subsidized rental flats vs 4.24% of the total population in Singapore living in one and two-room flats, the majority of which are heavily subsidized [29]). All subjects could be assigned to an exclusive segment for all segmentation schemes. A breakdown of study population segments based on our evaluated schemes is shown in Table 2. Some population segments were not represented by any subjects in this study and for clarity were not included in Table 2.

**Table 2:** Proportion of subjects in population segments based on different population segmentation schemes

Segmentation Scheme	Segment	Category	Mean age (years)	Percentage male (%)	EQ-5D Index	Count (% of total)	Perceived difficulty in meeting expenses (count, % of population segment)
Singapore	1	Healthy. Low RA	71.3	66.7	1.00	3 (0.3%)	0 (0.0%)
	2	Healthy. Moderate RA	69.5	73.0	0.945	137 (14.8%)	35 (25.5%)
	3	Healthy. High RA	69.3	66.7	0.932	3 (0.3%)	2 (66.7%)
	4	Asymptomatic Chronic Condition. Low RA	71.4	54.5	0.939	187 (20.2%)	42 (22.5%)
	5	Asymptomatic Chronic Condition. Moderate RA	71.2	62.6	0.878	230 (24.8%)	72 (31.3%)
	6	Asymptomatic Chronic Condition. High RA	72.8	53.3	0.880	15 (1.6%)	5 (33.3%)
	7	Symptomatic Chronic Condition. Low RA	69.8	63.6	0.820	33 (3.6%)	18 (54.5%)
	8	Symptomatic Chronic Condition. Moderate RA	72.4	52.0	0.634	98 (10.6%)	48 (49.0%)
	9	Symptomatic Chronic Condition. High RA	78.1	51.7	0.443	29 (3.1%)	11 (37.9%)
	10	Long Course of Decline. Low RA	70.0	51.4	0.874	35 (3.8%)	13 (37.1%)
	11	Long Course of Decline. Moderate RA	71.2	60.2	0.748	123 (13.3%)	48 (39.0%)
	12	Long Course of Decline. High RA	76.2	30.0	0.249	20 (2.2%)	10 (50.0%)
	13	Limited Reserve with Serious Exacerbations. Moderate RA	69.6	87.5	0.550	8 (0.9%)	5 (62.5%)
	14	Limited Reserve with Serious Exacerbations. High RA	74.3	42.9	0.614	7 (0.8%)	2 (28.6%)
Delaware	1	Adult & no chronic conditions	66.6	73.6	0.970	106 (11.4%)	28 (26.4%)
	2	Adult & 1 chronic condition	66.9	63.5	0.918	85 (9.2%)	25 (29.4%)
	3	Adult & 2+ chronic condition	67.5	62.1	0.884	269 (29.0%)	93 (34.6%)
	4	Adult & have mild mental health conditions (includes those with chronic illness)	66.7	55.9	0.697	143 (15.4%)	75 (52.4%)
	5	Adult & have severe mental health conditions (includes those with chronic illness)	67.1	56.3	0.690	48 (5.2%)	24 (50.0%)
	6	Elderly & no chronic conditions	81.7	62.1	0.908	29 (3.1%)	8 (27.6%)
	7	Elderly & 1 chronic condition	82.6	63.4	0.858	41 (4.4%)	9 (22.0%)
	8	Elderly & 2+ chronic condition	81.7	49.6	0.855	125 (13.5%)	24 (19.2%)
	9	Elderly & have mild mental health conditions (includes those with chronic illness)	80.8	51.6	0.582	64 (6.9%)	20 (31.3%)
	10	Elderly & have severe mental health conditions (includes those with chronic illness)	80.6	44.4	0.573	18 (1.9%)	5 (27.8%)
Lombardy	1	Healthy people	70.8	69.6	0.932	171 (18.4%)	43 (25.1%)
	2	People suffering from an acute event:	79.5	100.0	0.839	2 (0.2%)	1 (50.0%)
	3	People possibly affected by chronic disease or at early stage:	71.8	82.4	0.897	17 (1.8%)	8 (47.1%)
	4	People affected by only 1 chronic disease: consumed healthcare for chronic disease, continuous use of drugs,	66.4	60.9	0.890	110 (11.9%)	38 (34.5%)
	5	People affected by more than 1 chronic disease	70.4	57.7	0.794	562 (60.6%)	211 (37.5%)
	6	Elderly: 85 years or more	89.3	40.9	0.723	66 (7.1%)	10 (15.2%)
Northwest London	1	Mostly healthy adults < 75:	66.1	72.0	0.967	100 (10.8%)	25 (25.0%)
	2	Mostly health adults > 74:	80.2	69.7	0.939	33 (3.6%)	11 (33.3%)
	4	Adults < 75 with 1 or more LTCs:	66.7	61.6	0.858	430 (46.3%)	159 (37.0%)
	5	Elderly > 74 with 1 or more LTCs: same as group below but for those above the age of 75	80.4	54.5	0.835	213 (23.0%)	53 (24.9%)
	6	Adults and elderly people with SEMI: people aged above 16 who have a mental health problems	67.5	51.3	0.583	39 (4.2%)	23 (59.0%)
	7	Adults and elderly people with cancer: People aged above 16 who have any form and stage of cancer	71.4	45.5	0.801	44 (4.7%)	19 (43.2%)
	8	Adults and elderly people with severe physical disabilities	78.4	53.6	0.360	69 (7.4%)	21 (30.4%)

Evaluated population segmentation schemes were sourced from Singapore, Lombardy, Delaware and North-West London respectively. Total subject sample size: 928

*LTC* Long Term Conditions, *SEMI* Severe and Enduring Mental Illness

**Fig. 1:**
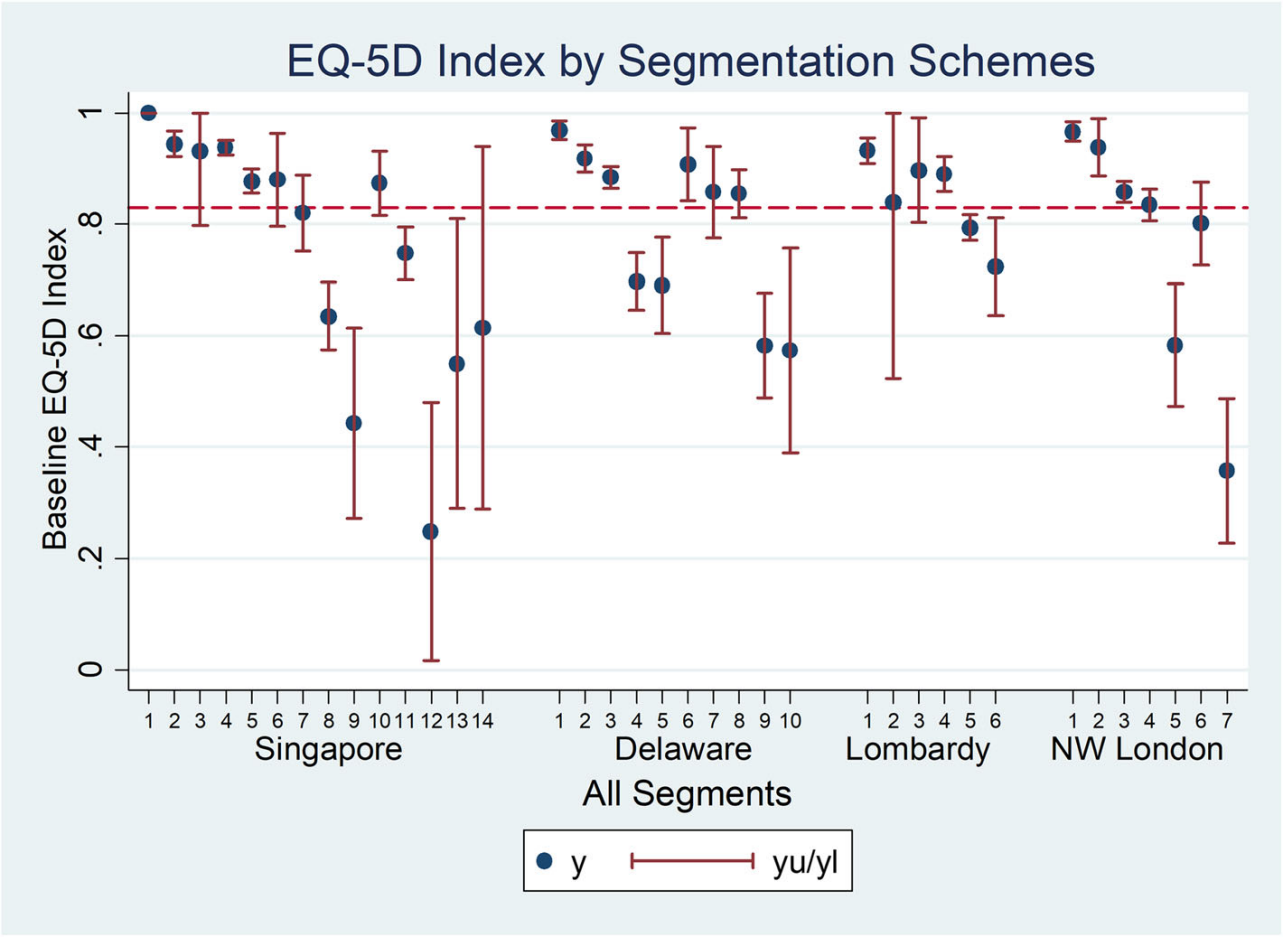
Health utility (EQ-5D Index score) by segment for 4 segmentation schemes

Health utility as assessed by EQ-5D Index was plotted in approximately ascending order of segment severity (Fig. 1). A total of 881 subjects (95%) had recorded data from which to calculate the EQ-5D Index scores. The mean EQ-5D index score was lower at baseline for subjects in more severe segments for each segmentation scheme. Overall, the mean EQ-5D score for all study subjects was 0.83. The population segment with the lowest mean EQ-5D index was Singapore category 12: Long course of decline with high RA with a mean EQ-5D of 0.249).

### Mortality and healthcare utilization by segment

**Fig. 2:**
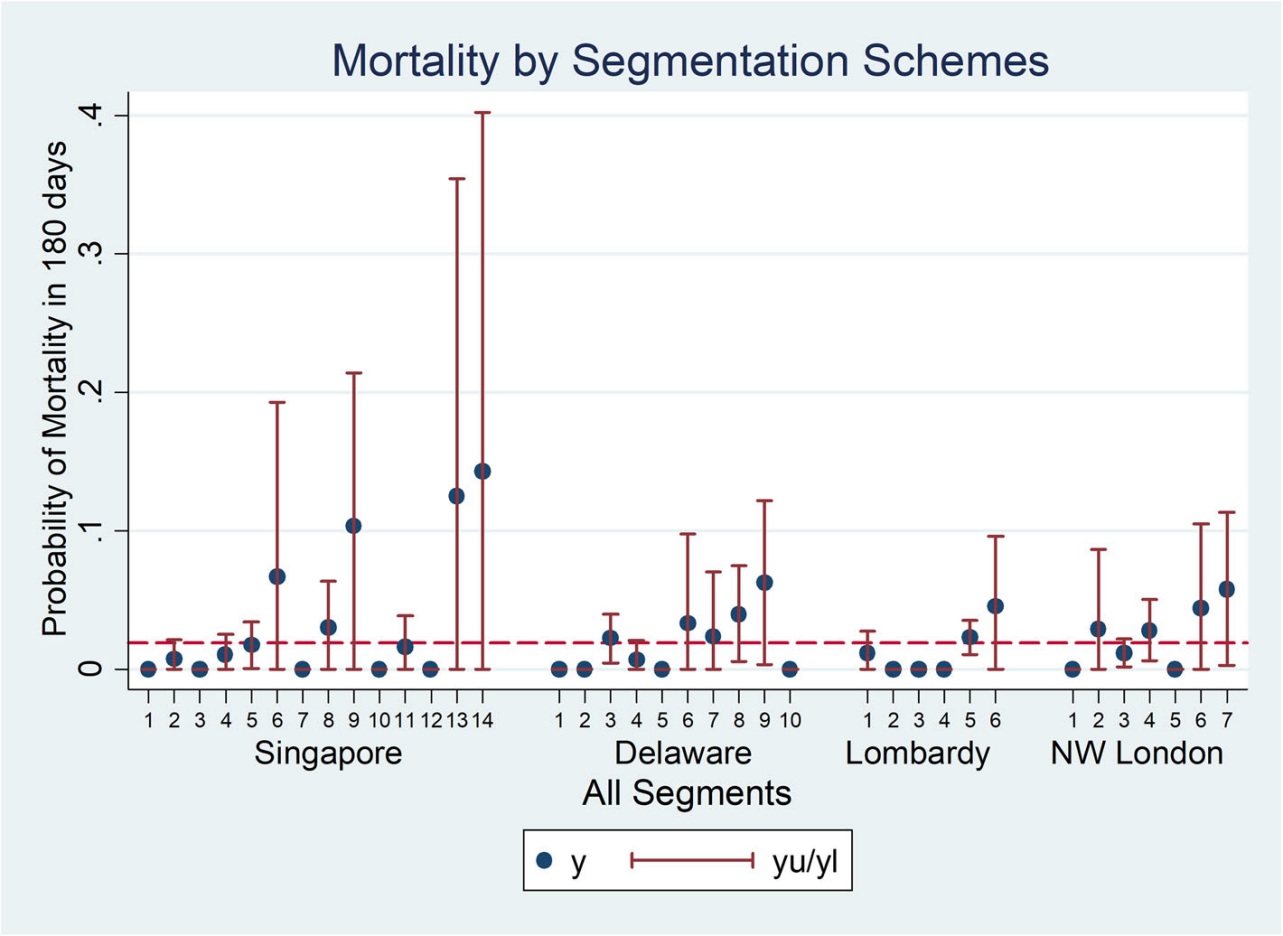
Mortality probability by segment for 4 segmentation schemes

Mortality rate is shown by segment in Fig. 2. Mortality rate for the overall population was 2%. For each segmentation scheme, there was a general trend of increasing mortality as population segment healthcare need increases. The population segment with the highest mean mortality risk was Singapore category 14: Limited Reserve with high Risk and Actionability (RA), with a 180-day mean mortality rate of 1 out of 7 individuals.

### Hospital admissions

**Fig. 3:**
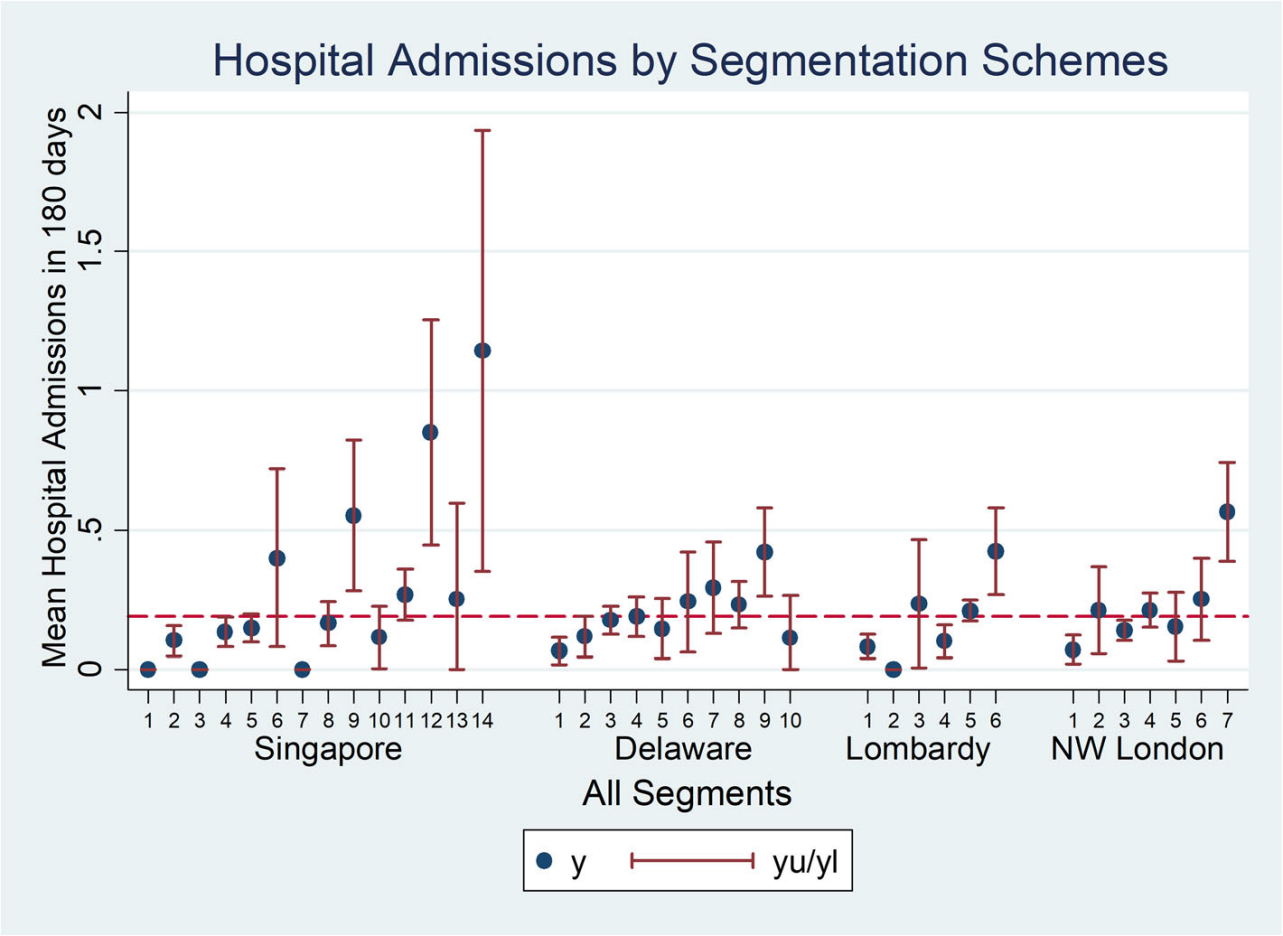
Mean number of hospital admissions by segment for 4 segmentation Schemes

Overall, the proportion of subjects with hospital admission within 180 days was 12.5% with a mean number of admissions per person in that period of 0.189 (Fig. 3). This rate tended to increase with severity of the health segment. The population segment with the largest mean number of admissions per person was Singapore category 14: Limited Reserve with high RA, with a mean number of visits of 1.14.

### ED visits

**Fig. 4:**
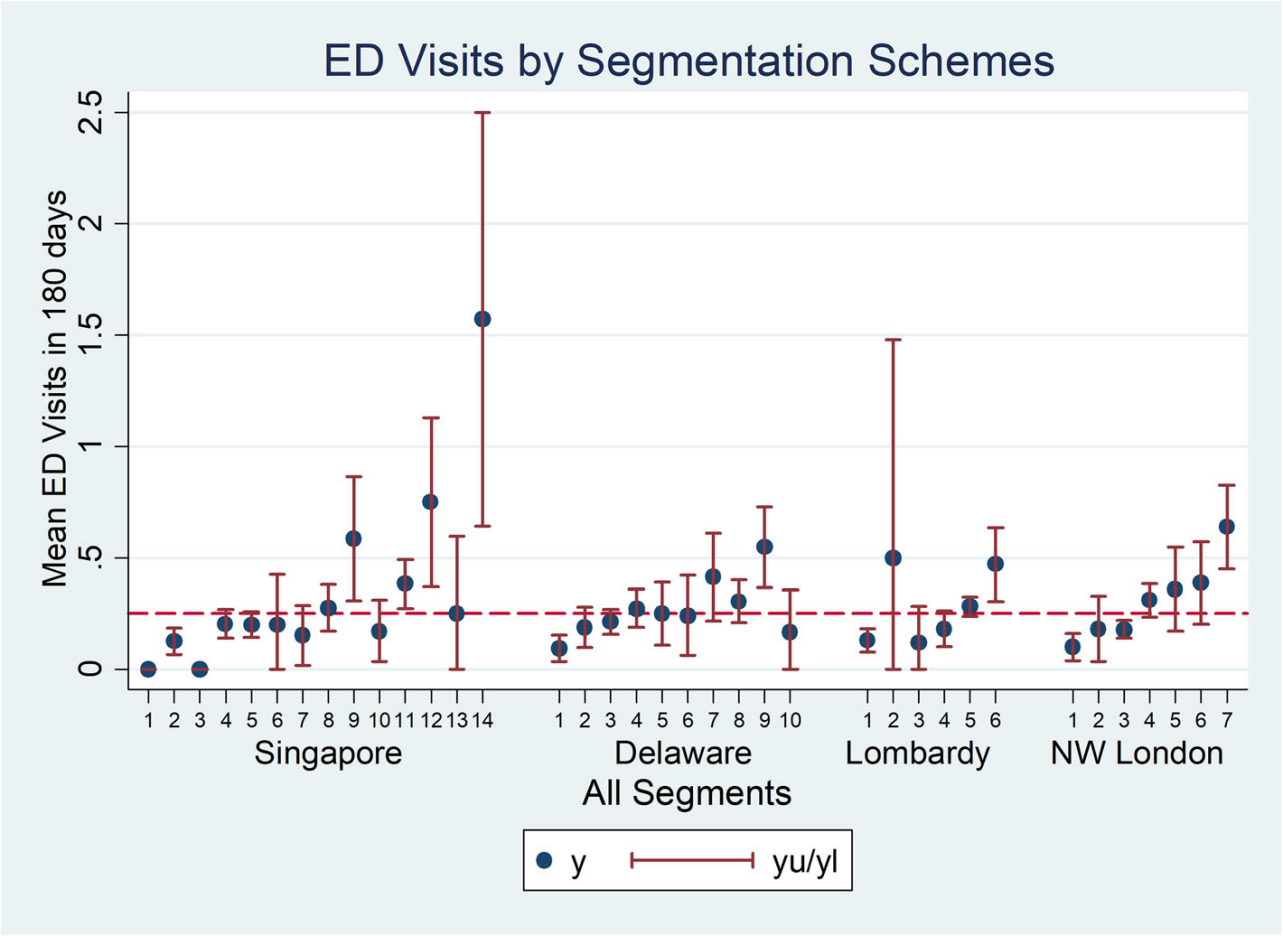
Mean number of ED visits by segment for 4 segmentation schemes

The overall proportion of subjects with ED visits over 180 days was 17.9% (Fig. 4). The mean number of ED visits per person in that period was 0.252 while the population segment with the highest mean ED visits Singapore category 14: Limited Reserve with high RA, with a mean number of visits of 1.57.

### SOC visits

**Fig. 5:**
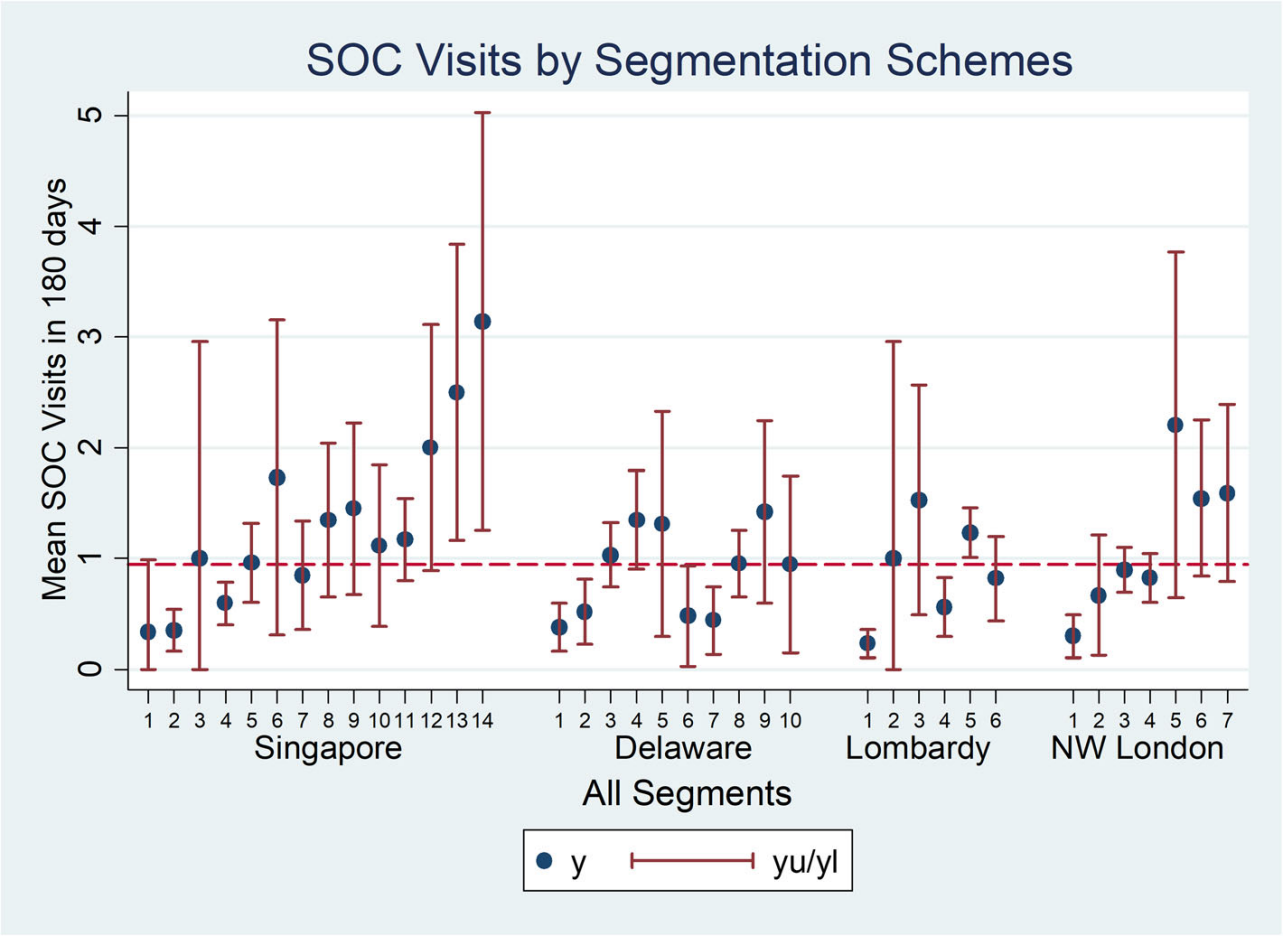
SOC visits by segment for 4 segmentation schemes

For all study subjects, 30.9% had SOC visits over 180 days (Fig. 5). The mean number of SOC visits for all study subjects was 0.945 visits while the population segment with the highest mean number of SOC visit was Singapore category 14: Limited Reserve with high RA, with a mean number of visits of 3.14.

### Test of statistical independence between population segments and outcomes

Based on the test results on Table 3, we observe that there is a statistically significant relationship between all 4 population segments and the outcomes of ‘any hospital admission’, ‘any ED visit’, and ‘any SOC visit’. Meanwhile for the outcome of mortality, we only observe a statistically significant relationship with the Singapore population segmentation scheme.

**Table 3:** Pearson’s Chi-squared tests between population segmentation schemes and healthcare outcomes in 180 days

Pearson’s Chi-squared test	Any SOC visit	Any ED visit	Any hospital admission	Any mortality
Singapore	65.1 ***	50.2 ***	57.3 ***	27.3 *
Delaware	28.8 **	22.7 **	27.4 **	15.8
Lombardy	50.3 ***	17.4 **	27.6 ***	5.85
Northwest London	33.0 ***	48.8 ***	47.5 ***	12.2

*p < 0.05, ** *p* < 0.01, *** *p* < 0.001

## Discussion

In a community at high risk for poor health outcomes based on factors such as age and socioeconomic status, we applied 4 different segmentation schemes purported to indicate health care need, based on an instrument fielded by non-medically trained surveyors. The survey had a high response rate (70%). All subjects could be assigned an exclusive segment for each segmentation scheme. Individuals in more severe segments tended to have lower quality of life as assessed by the EQ-5D Index for health utility. Quality of life is an important measure of overall patient well-being as it serves to quantify the impact of chronic disease [30]. All population segmentation schemes were observed to exhibit an ability to differentiate different levels of healthcare utilization with the most extreme outcomes for Singapore category 14: Limited Reserve with high Risk and Actionability. The Singapore segmentation categories had a statistically significant association with mortality, though all schemes demonstrated a trend.

More detailed examination provides some insight into the reasons for the ability of segmentation schemes generally to differentiate groups with higher mortality and health service utilization. Mortality risk tends to be higher among individuals with severe medical issues. Population segments defined by the presence of significant physical disabilities (e.g., Singapore categories 6, 9, 12, 14, North-West London category 7), cancer diagnosis (e.g., North-West London category 6), and elderly individuals (e.g., Delaware categories 6, 7, 8, 9, Lombardy category 6, North-West London categories 2,4) tend to have higher mortality risk compared to the other population segments. A similar pattern is seen for hospital admissions. However, for ED visits, the higher rates were primarily associated with segments defined by advanced age; these segments are Delaware categories 6, 7, 8, 9 (age 75 years and above), Lombardy category 6 (age 85 years and above) and North-West London categories 2 and 4 (age 75 years and above). Note Delaware category 10 (Elderly & have severe mental health conditions, including those with chronic illness) is an exception with individuals assigned to this segment having below average mean hospital admission rate. The difference between the patterns seen for mortality and hospital admission from ED visits may be attributable to the fact that ED visits are often patient initiated while a hospital admission event typically requires a medical professional’s judgment that an admission is indicated given the severity of a medical issue. Also, in many instances ED visits reflect unmet service needs [31, 32]. As seen in Fig. 4 for the Singapore categories, we observe that higher RA level, indicating high ADL needs and home nursing needs, consistently corresponds to higher ED utilization for all categories of medical needs (i.e., Global Impression).

Comparing Figs. 3 and 4, prospective ED utilization patterns closely mirror that of hospital admission utilization except for Lombardy segmentation category 2 ‘people suffering from an acute event’ and 3 ‘people possibly affected by chronic disease or at an early stage’. This suggests that individuals who have suffered an acute illness but do not have other chronic illnesses otherwise tend to not get readmitted to hospital as their conditions recover. However, in contrast, individuals whose diseases are at an early stage but may not have been properly evaluated are more likely to receive hospital admission due to exacerbation of their illnesses. For Lombardy categories, we observe the largest mean and confidence interval for ED utilization from category 2 ‘people suffering from an acute event’. With an acute illness such as a fracture, some of these subjects may have poor social support [33] and poor familiarity with coping strategies, thus leading to more ED visits for resolution of issues related to the acute illness. Finally, for North-West London categories, we observe that subjects in category 7 have the highest mean ED visit rate. This population segment suffers from severe physical disability such as ADL deficits thus may have less physical resilience [34] which contributes to the relatively high rate of ED utilization. Specialist outpatient clinic visits tend to be correlated with the number of different specialists being seen by a patient which in turn can be a proxy indicator of medical complexity or multiple comorbid conditions. That said, it is notable that non-elderly individuals with physical and mental ailments in this study tend to have more SOC visits than elderly individuals (see for example Delaware categories 5 and 10). Higher healthcare utilization may be related to socioeconomic factors such as perceived income adequacy as Delaware category 5 individuals had 50.0% of individuals reporting difficulty meeting expenses compared to Delaware category 10 which had 27.8%.

### Comparison with studies on similar segmentation schemes

While there are other studies of the practicality and predictive validity of various segmentation schemes, this study is unique in its application of the schemes on a community survey administered by non-medically trained personnel. Notably, the Lombardy [12], Delaware [11] and North-west London [13] segmentation schemes were originally developed to be applied to data sourced from electronic medical records. Also, while the Singapore scheme was developed to map groups of individuals to similar sets of health and health related social service needs, the other schemes used needs as a framework for classification, but focused on the prediction of per capita medical spending. None of the schemes, including the one developed in Singapore, has been evaluated with regard to the ability to predict responsiveness to interventions. Such demonstrated responsiveness would be the cardinal feature of an ideal segmentation scheme.

### Implications

The implications of this work should be considered in the context of the broader effort to develop a practical approach to segmenting a heterogenous population into parsimonious groupings with similar need, for the purpose of meeting needs effectively and efficiently. Existing healthcare needs-based population segmentation schemes can be classified as either expert or data driven. Expert based tools [5, 15–17, 35] have the advantage of being more clinically intuitive while data driven tools [36–38] have the advantage of being more objective as the population segments are generated using statistical clustering methods such as latent class analysis and hierarchical clustering. Tools that utilize data collected in EHR have the additional advantage of feasibility to segment large populations without the need for additional data collection. Regardless of how they were developed, both types of tools must be *practical* (i.e. require feasible data collection and produce a parsimonious number of segments), have demonstrable *external validity* (i.e. generalizable to populations other than the one used for tool development), and show *responsiveness* to specific package of health services (i.e. serve as a meaningful guide to efficient resource allocation)*.*

In this context, the 4 segmentation schemes evaluated are practical in terms of ease of data collection using a community-based survey. However, to the extent that some tools are intended to be used with an EHR which include complete inpatient and outpatient data, practical application will be limited in jurisdictions with less than comprehensive EHRs. In addition, all tools evaluated here produce a tractable number of segments, ranging from 6 to 14. Notably, the Johns Hopkins’ ACG, which was not assessed in this study, produces up to 92 segments [10].

Finally, we found that all evaluated segmentation schemes, including the non-Singapore ones, exhibited an ability to group patients into groups with relatively distinct healthcare patterns of health and utilization. This demonstrates predictive validity of all 4 segmentation schemes and *external validity* for the non-Singapore schemes.

As noted, a cardinal feature of a functionally useful segmentation scheme is *responsiveness*: its ability to predict change in health or utilization associated with specific packages of health services. This can be evaluated by comparing needs indicated by segment membership to actual services received and to assess whether concordance predicts better outcomes. To alleviate confounding due to the tendency of people in different segments to have substantially different propensities to receive certain services, responsiveness can also be evaluated in the context of a clinical trial.

In a more preliminary way, responsiveness can be assessed in terms of the degree to which the scheme has the potential to guide service allocation (i.e., actionability). This feature distinguishes segmentation schemes that are needs-based (i.e., designed to indicate prototypical services expected to reduce risk) from schemes that are risk-based (predict individuals likely to have poor health outcomes or high health care utilization) [39]. In this regard, the Singapore scheme is distinctive as it was specifically developed to be indicative of services expected to reduce risk of poor outcomes. Compared to the other population segmentation schemes, the Singapore scheme appears to capture the largest number of actionable health and health-related social needs (see Additional file 5 for comparison table).

### Strengths

This study has several strengths. First, it is pragmatic, having been performed in a community that faces practical challenges for the health system. It includes individual who would not have been assessed by use of EHR because they did not have contact with the health system holding that individuals records, either because they successfully avoided medical attention, or used services in other health venues not part of the EHR ecosystem. Second, the study provides an opportunity to evaluate the Singapore segmentation scheme relative to other schemes using a common data set.

### Limitations

There are several limitations to this study. Firstly, not all population segments could be evaluated fully in our analysis given that the community survey did not have all the required variables and subject types (subjects in this study were low-income elderly individuals aged 60 years and above) for fully characterizing all population segments. Specifically, 10 segments from the Delaware scheme were not evaluated as the inclusion criteria for the survey were subjects at least 60 years old, 2 segments from the Lombardy scheme were not evaluated due to a similar reason as well as the lack of utilization data spanning the previous 3 years, 3 segments from the North-West London scheme were not evaluated as the survey did not capture information on alcohol/drug dependency, learning difficulties, as well as an absence of any subjects with dementia but no physical disabilities, and finally, 4 segments from the Singapore scheme were not evaluated given that no subject with limited reserve but low RA exists in the study and the survey did not capture information on end-of-life medical conditions. Secondly, the external validity of our results may be limited by the fact that our study subjects comprise low income elderly individuals aged 60 and above. Individuals with low income may not readily seek healthcare as they have limited financial resources or may be more prone to overutilize services that are subsidized. Thus, our results may not generalize readily to populations from other age group and income levels. Thirdly, some of the population segments had a relatively small number of subjects which resulted in wide confidence intervals that tend to overlap between population segments. For example, Singapore scheme categories 13 and 14 both contained less than 10 subjects and is noted to have relatively large and overlapping confidence intervals for most of the evaluated outcomes. To evaluate mortality and health care use, a competitive risk analysis would have been ideal and should be pursued in future studies should time-to-event data be available. Finally, the existing models still has areas for improvement in terms of its ability to predict mortality.

### Future work

One line of future work should aim to clarify issues resulting from the limitations noted above. This would include assuring that in a future prospective community-based study captures all variables needed by the various segmentation schemes. Future studies should oversample individuals likely to fall into relatively uncommon segments in order to establish adequate power to assess differences in outcomes. A second, crucial area, for study is to empirically evaluate responsiveness: how meeting service needs impact transition rates to worse health needs states. As this study has demonstrated the feasibility of utilizing patient reported variables for purposes of segmentation, longitudinal follow-up data gathering exercises may potentially be performed through telephone interviews which are logistically easier to execute compared to in-person interviews. Alternatively, core data needed for population segmentation can be embedded into the EHR and clinical workflow to allow ongoing assessment of transition rates between segments and the determinants of those rates. The responsiveness of segmentation schemes can be further addressed by incorporating them into evaluations of integrated packages of health services designed for specific population segments to allow more precise evaluation of the efficacy of these services for improving health outcomes. A third line of future work is to replicate this study in different countries, including additional population segmentation schemes as appropriate in order to gain insights about the nature of health service needs globally, and how individuals in different countries respond to interventions. Lastly, in order to continually improve the segmentation scheme’s ability to predict variability in relevant healthcare outcomes and responsiveness to types and modes of service, future work can include identification of combinations of predictive variables that provide better guides to service allocation, in the spirit of a “learning health system” [40].

## Conclusion

It is practical to assign individuals to healthcare needs-based population segments through community surveys by non-medically trained personnel. The resulting segments for all 4 schemes evaluated in this way have an ability to predict health outcomes and utilization over the medium term (180 days), with significant overlap for some segments. Healthcare needs-based segmentation schemes which are designed to guide action holds particular promise for promoting efficient allocation of services to meet the needs of salient population groups. Further evaluation is needed to determine if these schemes also predict responsiveness to interventions to meet needs implied by segment membership.

## Supplementary information

**Additional file 1.** Simple Segmentation Tool (SST) V2. Checklist designed to capture variables which enable healthcare need based population segmentation using the SST framework.

**Additional file 2.** Respondent questionnaire. Questionnaire administered by research personnel to subjects in this study. Designed to be bilingual given the high prevalence of Chinese respondents in the target community.

**Additional file 3.** Mapping scheme. Detailed segmentation base of population segmentation schemes evaluated in this study.

**Additional file 4.** STROBE checklist for cohort studies. STROBE Statement – Checklist of items that should be included in reports of cohort studies.

**Additional file 5.** Comparison of actionability. Comparison of all 4 segmentation schemes in this study in terms of number and type of healthcare need type variables utilized as input to the respective schemes.

## Data Availability

Datasets used and/or analyzed during the current study are available from the corresponding author on reasonable request.

## Abbreviations

ADL: Activity of daily living
AMT: Abbreviated mental test
ED: Emergency department
eHints: Electronic health intelligence system
EHR: Electronic health record
EQ-5D: EuroQol 5 dimensions
HRQOL: Health related quality of life
LTC: Long term conditions
RA: Risk and actionability
SEMI: Severe and enduring mental illness
SOC: Specialist outpatient clinics
SST: Simple segmentation tool
