# Supplementary material for: Do healthcare needs-based population segments predict outcomes among the elderly? Findings from a prospective cohort study in an urbanized low-income community
Source: BMC Geriatr. 2020 Feb 27;20:78. doi: 10.1186/s12877-020-1480-9 (PMC7045405; doi:10.1186/s12877-020-1480-9)
Supplement: Supplementary file 2 — Additional file 2. Respondent questionnaire. Questionnaire administered by research personnel to subjects in this study. Designed to be bilingual given the high prevalence of Chinese respondents in the target community. [file 12877_2020_1480_MOESM2_ESM.doc]

| **SECTION A: BASIC ATTRIBUTES AND FAMILY MAKE-UP**  **A部分：基本属性和家庭构成** | | |
| --- | --- | --- |
| **READ OUT:** You will be asked a number of questions during this survey. You do not have to respond to any questions that you feel uncomfortable answering. Also, please rest assured that all of your responses will be kept strictly confidential and will not be shown to or shared with anyone not connected with this survey. | | |
| **读出：**本次调查会问您很多问题。您无需每个都回答，若您感觉不适，可拒绝作答。同样，请放心，我们会对您的回答严格保密，不会公开或与任何与本调查无关的人分享。  **First, you will be asked questions about yourself.**  **首先，会询问您的个人信息。** | | |
|  | | |
| A1 | What is your nationality?  您的国籍？  Singapore Citizen  新加坡公民    Singapore Permanent Resident (please state nationality: __________________________)  新加坡永久居民（请注明国籍：_________________________） | Code  1  2 |
| A2 | What is your date of birth? How old are you now?  您的出生日期？您目前年龄？  ⬜⬜ Day 日 ⬜⬜ Month 月 ⬜⬜ Year 年 ⬜⬜⬜ Years Old 岁 |  |
| A3 | Record gender of respondent.  您的性别？  Male 男  Female 女 | Code  1  2 |
| A4 | What is your race?  您的种族？  Chinese 华族  Malay 马来族  Indian 印度族  Others, please specify: ____________________________ | Code  1  2  3  4 |
| A5 | What is your religion?  您的宗教？  Christianity  基督教  Buddhism / Taoism  佛教/道教  Islam  伊斯兰教  Hinduism  印度教  Others, please specify: ________________  其他，请注明：____________________________  No religion  无宗教 | Code  1  2  3  4  5  6 |
| A6 | What is your current marital status?  您当前的婚姻状态？  Married (includes being separated from the spouse due to one’s spouse being hospitalized, living in an institution, or living in another area for business reasons or to take care of others)  已婚（包括由于配偶住院、住进疗养院或因工作或照顾他人而居住别处而分离）  Widowed  丧偶  Separated from spouse  分居  Divorced  离异  Never married  未婚 | Code  1  2  3  4  5 |
| A7 | What is the highest education level you have completed?  您已完成的最高教育程度？  No formal education  未接受过正式教育  Primary  小学  Secondary  初中  Vocational / ITE  职中/技校  Junior College / Poly  大专/理工学院  University and above  大学及以上  Don’t know / can’t remember  不详/忘记 | Code  1  2  3  4  5  6  7 |
| A8 | Are you currently working?  您目前在工作吗？  Working full-time 全职  Working part-time 兼职  Retired and/or not working 退休和/或未工作  Homemaker 主妇 | Code  1  2  3  4 |
| A9 | **[SHOWCARD A9]** What are the main reasons you moved to Chin Swee (maximum 3)?  您搬到振瑞小区的主要原因（最多3个）？  Financial reasons  经济原因  To receive help from family  为了获得家人帮助  To provide help to family  为了帮助家人  To be looked after  为了受照看  Want to be near my child(ren) or family  想要靠近孩子或家人  My husband/wife wanted to  丈夫/妻子要求  My husband/wife passed away  丈夫/妻子去世  House allotted here by the government  因为政府分配房  My children requested me to move out  我的孩子要求我搬出去  Others, please explain: ________________________________________________  其他，请说明：________________________________________________  Not sure  不确定 | Code  1  2  3  4  5  6  7  8  9  10  11 |
| A10 | How many persons are there living in your household? (Including yourself, maid and those who are temporarily hospitalized, children who are living in hostel within Singapore, and household members who are currently serving National Service/Reservist training. __________________ (number)  您家里住了几个人？（包括您本人、佣人和暂时住院人员、住在学校宿舍的子女以及正在服兵役/接受预备役军人训练的家人。__________________（人数）  [*Interviewer: This included all who live in the household presently, as well as those who have this address recorded in their NRIC,* ***excluding tenants****].*  *[采访人员：包括目前所有住在该家庭里的人员以及身份证登记在此的人员，不包括租户]。* | |
| A11 | Please tell us how many children you have who are currently alive? __________________ (number)  您目前尚在世的子女人数？__________________（人数） | |

| **SECTION B: HEALTH STATUS & PHYSICAL DISABILITIES / LIMITATIONS**  **B部分：健康状态和残疾/行动不便** | | | |
| --- | --- | --- | --- |
| **The next questions will be regarding health.**  **接下来的问题有关健康。** | | | |
| B1 | **[SHOWCARD B1]** In general, how would you describe your state of health?  整体上，您的健康状况如何？  Excellent 非常好  Very good 很好  Good 好  Fair 一般  Poor差  Not sure / Don’t know 不确定/不知道 | | Code  1  2  3  4  5  99 |
| B2 | **[SHOWCARD B2]** The next question is related to vision. With your glasses or contact lenses, if you wear them, how is your vision?  下一道问题与您的视力有关。若您带着眼镜或隐形眼镜，您的视力如何？  Excellent 非常好  Very good 很好  Good 好  Fair 一般  Poor差  Loss of vision (no vision) in both eyes 双眼失明（看不见）  Not sure/ Don’t know 不确定/不知道 | | Code  1  2  3  4  5  6  99 |
| B3 | **[SHOWCARD B3]** The following question is related to hearing ability. If you use hearing aids, please respond to this question based on your hearing ability when you wear them. How is your hearing?  下一道问题与您的听力有关。若您使用助听器，则根据其戴着助听器时的听力能力回答此题。听力如何？  Excellent 非常好  Very good 很好  Good 好  Fair 一般  Poor差  Not able to hear in both ears 双耳失聪  Not sure/Don’t know 不确定/不知道 | | Code  1  2  3  4  5  6  99 |
| B4 | In the last year, have you lost more than 5 kilograms of body weight unintentionally (i.e., not due to dieting or exercise)?  在过去的一年里，您是否有无意地减重超过五公斤？也就是说不是因节食或运动导致的体重下降。  Yes 是的  No 没有  Not sure/Don’t know 不确定/不知道 | | Code  1  2  99 |
| B5a | **[ROUTING QNS]** Have you fallen down in the last one year?  您在过去一年跌倒过吗？  Yes 是的  No 没有  Not sure/ Don’t know 不确定/不知道 | Code  1  2  99 | Route  B6a  B6a |
| B5b | How many times have you fallen in the last one year? ___________  您在过去一年跌倒次数？___________  Don’t know / Can’t remember 不详/忘记 | | Code  99 |
| B5c | In that fall/ In any of these falls, did you injure yourself seriously enough to need medical treatment?  该（每）次跌倒您是否严重受伤需要就医？  Yes 是的  No 没有  Not sure/ Don’t know 不确定/不知道 | | Code  1  2  99 |
| B6a | **[ROUTING QNS]** If there is a free exercise program to improve your balance and gait in your neighbourhood, would you be willing to attend?  若在您附近有一个针对改善您的平衡和步态的免费运动项目，您愿意参加吗？  Yes 是的  No 没有  Not sure/Don’t know 不确定/不知道 | Code  1  2  99 | Route  B6b  B7  B7 |
| B6b | How many times a week are you willing to attend? _____________  您一周愿意参加几次？_____________  Not sure/Don’t know 不确定/不知道 | | Code  99 |

| **The next questions concern your ability to perform daily activities.**  **接下来的问题是关于您的日常活动能力。**  **[ROUTING QNS]** ***[Interviewer: Ask a-f, repeating (i) and (ii) for each appropriate.]*** | | | | | | | |
| --- | --- | --- | --- | --- | --- | --- | --- |
| B7 | **[SHOWCARD B7]** | | | | | | |
| **Activities 活动** | | 1. **(i) Do you find it difficult to perform this activity alone without the assistance of a person or assistive device due to your health or physical state**   **(i) 以您（参与者姓名）的健康或体能状况，在没有他人帮助或任何辅助器的协助下自行做这个活动，会有困难吗？** | **Code** | **(ii) Do you find it difficult to perform this activity alone without the assistance of a person or assistive device due to your health or physical state (ii) 您（参与者姓名）自行做这个活动时有多困难？** | | | **Code** |
| a. | Take a bath/shower  洗澡/淋浴 | Difficult 困难 🡪 Go to (ii)  Not difficult 不难  Not sure 不确定 | 1  2  3 | Somewhat difficult 有难度  Very difficult 很难  Unable to perform 不能进行  Not sure 不确定 | | | 1  2  3  4 |
| b. | Dress up  穿衣 | Difficult 困难 🡪 Go to (ii)  Not difficult 不难  Not sure 不确定 | 1  2  3 | Somewhat difficult 有难度  Very difficult 很难  Unable to perform 不能进行  Not sure 不确定 | | | 1  2  3  4 |
| c. | Eat  吃饭 | Difficult 困难 🡪 Go to (ii)  Not difficult 不难  Not sure 不确定 | 1  2  3 | Somewhat difficult 有难度  Very difficult 很难  Unable to perform 不能进行  Not sure 不确定 | | | 1  2  3  4 |
| d. | Stand up from a bed/chair, sitting down on a chair  起床/从椅子上起身，坐下 | Difficult 困难 🡪 Go to (ii)  Not difficult 不难  Not sure 不确定 | 1  2  3 | Somewhat difficult 有难度  Very difficult 很难  Unable to perform 不能进行  Not sure 不确定 | | | 1  2  3  4 |
| e. | Walk (around the house)  步行（房子周围） | Difficult 困难 🡪 Go to (ii)  Not difficult 不难  Not sure 不确定 | 1  2  3 | Somewhat difficult 有难度  Very difficult 很难  Unable to perform 不能进行  Not sure 不确定 | | | 1  2  3  4 |
| f. | Go outside (leave the house)  外出（离开房子） | Difficult 困难 🡪 Go to (ii)  Not difficult 不难  Not sure 不确定 | 1  2  3 | Somewhat difficult 有难度  Very difficult 很难  Unable to perform 不能进行  Not sure 不确定 | | | 1  2  3  4 |
| g | Use the sitting toilet  使用坐便器 | Difficult 困难 🡪 Go to (ii)  Not difficult 不难  Not sure 不确定 | 1  2  3 | Somewhat difficult 有难度  Very difficult 很难  Unable to perform 不能进行  Not sure 不确定 | | | 1  2  3  4 |
| B8 | [Interviewer Record: Did the participant report any difficulty in any of the activities listed in B7?]  【调查员请记录B7中受访者是否有需要帮助的项目】？  Yes 是的  No 没有 | | | | Code  1  2 | Route  B9  B11 | |
| B9 | Who primarily helps you perform these activities?  主要是谁帮助您进行以上活动？  No one  没有人  Live-in family member (Specify: ___________________)  同居的家人（说明：___________________）  Non- co-resident family member  未住在一起的家人  Maid / Foreign Domestic Worker  佣人/外籍帮佣  Not sure  不确定  Other (Specify: ___________________)  其他（请注明：____________________________） | | | | | Code  1  2  3  4  5  6 | |
| B10 | Do these difficulties limit your social interaction?  这些困难限制了您的社交吗？  Yes 是的  No 没有  Not sure 不确定 | | | | | Code  1  2  3 | |

| B11 | **[SHOWCARD B11] Choose the scoring point for the statement that most corresponds to your current functional ability for each task**  **（i）请选择最符合您在执行以下任务时的能力水平的得分** | | | |
| --- | --- | --- | --- | --- |
| **Activities活动** | | |  |  |
| a. | Ability to use telephone  使用电话的能力 | Operates telephone on own initiative; looks up and dials numbers  自主操作电话; 查找并拨打电话号码  Dials a few well-known numbers 拨打几个常用的号码  Answers telephone but does not dial 能接电话但不能打电话  Does not use telephone at all 不用电话 | 1  2  3  4 | Code  1  1  1  0 |
| b. | Shopping  购物 | Takes care of all shopping needs independently 自己独立买东西  Shops independently for small purchases 可独立进行少量采购  Needs to be accompanied on any shopping trip 需要在他人陪同下购物  Completely unable to shop 完全不能购物 | 1  2  3  4 | Code  1  0  0  0 |
| c. | Food  Preparation  做饭 | Plans, prepares, and serves adequate meals independently  独立计划、制作并提供足够的饭菜  Prepares adequate meals if supplied with ingredients  若提供了食材，可制作足够的饭菜  Heats and serves prepared meals, or prepares meals but does not maintain adequate diet  加热已做好的饭菜或可以做饭但不能维持足够的饮食  Needs to have meals prepared and served  需要别人制作并提供饭菜 | 1  2  3  4 | Code  1  0  0  0 |
| d. | Housekeeping  家务 | Maintains house alone or with occasional assistance (e.g. “heavy work domestic help”)  能独自处理家务或偶尔需要帮助（如“繁重的家务需要家政人员的帮助”）  Performs light daily tasks such as dishwashing, bed making  完成较轻的日常工作，如洗碗和铺床  Performs light daily tasks but cannot maintain acceptable level of cleanliness  能完成较轻的日常工作，但不能保持令人满意的清洁度  Needs help with all home maintenance tasks  所有家务均需他人帮助  Does not participate in any housekeeping tasks  不参与任何家务 | 1  2  3  4  5 | Code  1  1  1  1  0 |
| e. | Laundry  洗衣 | Does personal laundry completely 完全自己洗衣  Launders small items; rinses socks, stockings, etc. 洗小物品；洗袜子等  All laundry must be done by others 所有衣物均由他人清洗 | 1  2  3 | Code  1  1  0 |
| f. | Mode of transportation  交通方式 | Travels independently on public transportation or drives own car  独立乘坐公共交通或自己开车  Arrange own travel via taxi, but does not otherwise use public transportation  自己乘搭计程车，但不能使用其他公共交通  Travels on public transportation when assisted or accompanied by another  在他人帮助或陪同下能乘坐公共交通  Travel limited to taxi or automobile with assistance of another  仅能在他人帮助下乘坐计程车或汽车  Does not travel at all  不能出行 | 1  2  3  4  5 | Code  1  1  1  0  0 |
| g. | Responsibility for own medications  服药能力 | Is responsible for taking medication in correct dosages at correct time  能在正确的时间服用正确剂量的药物  Takes responsibility if medication is prepared in advance in separate dosages  若药物剂量已提前分好，可自行服药  Is not capable of dispensing own medication  不能自己分药 | 1  2  3 | Code  1  0  0 |
| h. | Ability to handle finances  可自己理财 | Manages financial matters independently (budgets, writes checks, pays rent and bills, goes to bank), collects and keep tracks of income  独立管理财务（预算、开支票、付租金和账单、去银行）、领取并记录收入  Manages day-to-day purchases, but needs help with banking, major purchases, etc.  管理日常购物，但需要他人协助办理银行业务和大件购物等  Incapable of handling money  不能理财 | 1  2  3 | Code  1  1  0 |

| B12 | **[ROUTING QNS]** Do you require help in any of the activities listed in B11?  [请问您是否需要任何帮助来执行以上的项目？]  Yes 是的  No 没有 | Code  1  2 | Route  B13  B15 |
| --- | --- | --- | --- |
| B13 | Who primarily helps you perform these activities?  主要是谁帮助您进行以上活动？  No one  没有人  Live-in family member (Specify: _______________) [Interviewer: record relationship of family member, i.e. son, daughter, spouse, etc.]  同居的家人（注明：_______________）[采访人员：记录家庭成员关系，即子女、配偶等]  Non co-resident family member  未住在一起的家人  Maid / Foreign Domestic Worker  佣人/外籍帮佣  Other (Specify: ___________________)  其他（请注明：____________________________）  Not sure/Don’t know  不确定/不知道  Refused  拒绝作答 | | Code  1  2  3  4  5  99  77 |
| B14 | Do these difficulties limit your social interaction?  这些困难限制了您的社交吗？  Yes 是的  No 没有  Not sure/Don’t know 不确定  Refused 拒绝作答 | | Code  1  2  99  77 |

| **B15 [SHOWCARD B15] The next questions will be about experience with physical ailments that you have had.**  ***[Interviewer: Ask a-t, repeating (i), (ii), (iii) and (iv) for each as appropriate.]***  **接着的问题是关于您（参与者姓名）是否有患上或曾患上的疾病。**  ***[采访人员：针对问题（i）、（ii）、（iii）和（iv），酌情重复询问a-t。]*** |
| --- |
| | **Ailment 疾病** | | **(i) Have you ever been diagnosed by a medical professional with:**  **(i) 是否有专业医生曾经诊断您患有：** | | | **(ii) Have you ever been treated with medicine or surgery for this condition?**  **(ii) 您是否就此情况接受过药物或手术治疗？** | | | **(iii) Have you been hospitalized or gone to A&E for this condition in the past six months?**  **(iii) 过去六个月内您是否因此情况住院或急诊？** | | | **(iv) Please state the number of times you have been hospitalized or gone to A&E for this condition in the past six months?**  **(iv) 请说明过去六个月内您因此情况住院或急诊次数？** | | | --- | --- | --- | --- | --- | --- | --- | --- | --- | --- | --- | --- | --- | | **Yes 是的** | **No没有** | **Not Sure不确定** | **Yes 是的** | **No**  **没有** | **Not Sure**  **不确定** | **Yes 是的** | **No 没有** | **Not Sure**  **不确定** | **A&E visit only**  **急诊** | **Hospital admission only**  **住院** | | a. | Heart attack, angina, myocardial infarction  心脏病、心绞痛、心肌梗死 | 1  ***[Go to (ii)]*** | 2 | 3 | 1  ***[Go to (iii)]*** | 2 | 3 | 1  ***[Go to (iv)]*** | 2 | 3 | **______times/次** | **______times/次** | | b. | Heart failure  心衰 | 1  ***[Go to (ii)]*** | 2 | 3 | 1  ***[Go to (iii)]*** | 2 | 3 | 1  ***[Go to (iv)]*** | 2 | 3 | **______times/次** | **______times/次** | | c. | Other forms of heart diseases  其他心脏疾病 | 1  ***[Go to (ii)]*** | 2 | 3 | 1  ***[Go to (iii)]*** | 2 | 3 | 1  ***[Go to (iv)]*** | 2 | 3 | **______times/次** | **______times/次** | | d. | Cancer (excluding skin cancer)  癌症（不包括皮肤癌） | 1  ***[Go to (ii)]*** | 2 | 3 | 1  ***[Go to (iii)]*** | 2 | 3 | 1  ***[Go to (iv)]*** | 2 | 3 | **______times/次** | **______times/次** | | e. | Cerebrovascular disease ( such as stroke)  脑血管疾病（如中风） | 1  ***[Go to (ii)]*** | 2 | 3 | 1  ***[Go to (iii)]*** | 2 | 3 | 1  ***[Go to (iv)]*** | 2 | 3 | **______times/次** | **______times/次** | | f. | High blood pressure  高血压 | 1  ***[Go to (ii)]*** | 2 | 3 | 1  ***[Go to (iii)]*** | 2 | 3 | 1  ***[Go to (iv)]*** | 2 | 3 | **______times/次** | **______times/次** | | g. | Diabetes  糖尿病 | 1  ***[Go to (ii)]*** | 2 | 3 | 1  ***[Go to (iii)]*** | 2 | 3 | 1  ***[Go to (iv)]*** | 2 | 3 | **______times/次** | **______times/次** | | h. | High blood cholesterol or lipids  高血胆固醇或高血脂 | 1  ***[Go to (ii)]*** | 2 | 3 | 1  ***[Go to (iii)]*** | 2 | 3 | 1  ***[Go to (iv)]*** | 2 | 3 | **______times/次** | **______times/次** | | i. | Dementia ***[ask only from proxy]***  老年矢志症[仅从代理人处了解] | 1  ***[Go to (ii)]*** | 2 | 3 | 1  ***[Go to (iii)]*** | 2 | 3 | 1  ***[Go to (iv)]*** | 2 | 3 | **______times/次** | **______times/次** | | **Ailment 疾病** | | **(i) Have you ever been diagnosed by a medical professional with:**  **（i）是否有专业医生曾经诊断您患有：** | | | **(ii) Have you ever been treated with medicine or surgery for this condition?**  **（ii）您是否就此情况接受过药物或手术治疗？** | | | **(iii) Have you been hospitalized or gone to A&E for this condition in the past six months?**  **（iii）过去六个月内您是否因此情况住院或急诊？** | | | **(iv) Please state the number of times you have been hospitalized or gone to A&E for this condition in the past six months?**  **（iv）请说明过去六个月内您因此情况住院或急诊次数？** | | | **Yes**  **是的** | **No没有** | **Not Sure不确定** | **Yes**  **是的** | **No**  **没有** | **Not Sure**  **不确定** | **Yes**  **是的** | **No 没有** | **Not Sure**  **不确定** | **A&E visit only**  **急诊** | **Hospital admission only**  **住院** | | j. | Chronic respiratory illness (e.g. asthma)  慢性呼吸系统疾病（如哮喘） | 1  ***[Go to (ii)]*** | 2 | 3 | 1  ***[Go to (iii)]*** | 2 | 3 | 1  ***[Go to (iv)]*** | 2 | 3 | **______times/次** | **______times/次** | | k. | Digestive illness (stomach or intestinal)  消化道疾病（胃肠） | 1  ***[Go to (ii)]*** | 2 | 3 | 1  ***[Go to (iii)]*** | 2 | 3 | 1  ***[Go to (iv)]*** | 2 | 3 | **______times/次** | **______times/次** | | l. | Renal/kidney or urinary tract ailments  肾或泌尿系统疾病 | 1  ***[Go to (ii)]*** | 2 | 3 | 1  ***[Go to (iii)]*** | 2 | 3 | 1  ***[Go to (iv)]*** | 2 | 3 | **______times/次** | **______times/次** | | m. | Ailments of the liver or gallbladder  肝或胆囊疾病 | 1  ***[Go to (ii)]*** | 2 | 3 | 1  ***[Go to (iii)]*** | 2 | 3 | 1  ***[Go to (iv)]*** | 2 | 3 | **______times/次** | **______times/次** | | n. | Joint pain, arthritis, rheumatism or nerve pain  关节痛、关节炎、风湿或神经痛 | 1  ***[Go to (ii)]*** | 2 | 3 | 1  ***[Go to (iii)]*** | 2 | 3 | 1  ***[Go to (iv)]*** | 2 | 3 | **______times/次** | **______times/次** | | o. | Chronic back pain  慢性背痛 | 1  ***[Go to (ii)]*** | 2 | 3 | 1  ***[Go to (iii)]*** | 2 | 3 | 1  ***[Go to (iv)]*** | 2 | 3 | **______times/次** | **______times/次** | | p. | Osteoporosis  骨质疏松症 | 1  ***[Go to (ii)]*** | 2 | 3 | 1  ***[Go to (iii)]*** | 2 | 3 | 1  ***[Go to (iv)]*** | 2 | 3 | **______times/次** | **______times/次** | | q. | Fractures of the hip, thigh and pelvis  臀部、大腿和骨盆骨折 | 1  ***[Go to (ii)]*** | 2 | 3 | 1  ***[Go to (iii)]*** | 2 | 3 | 1  ***[Go to (iv)]*** | 2 | 3 | **______times/次** | **______times/次** | | r. | Other fractures  其它骨折 | 1  ***[Go to (ii)]*** | 2 | 3 | 1  ***[Go to (iii)]*** | 2 | 3 | 1  ***[Go to (iv)]*** | 2 | 3 | **______times/次** | **______times/次** | | s. | Cataract  白内障 | 1  ***[Go to (ii)]*** | 2 | 3 | 1  ***[Go to (iii)]*** | 2 | 3 | 1  ***[Go to (iv)]*** | 2 | 3 | **______times/次** | **______times/次** | | t. | Glaucoma  青光眼 | 1  ***[Go to (ii)]*** | 2 | 3 | 1  ***[Go to (iii)]*** | 2 | 3 | 1  ***[Go to (iv)]*** | 2 | 3 | **______times/次** | **______times/次** | | u. | Others (Specify:________________)  其他（注明: _______________________） | 1  ***[Go to (ii)]*** | 2 | 3 | 1  ***[Go to (iii)]*** | 2 | 3 | 1  ***[Go to (iv)]*** | 2 | 3 | **______times/次** | **______times/次** | |

| B16 | **[SHOWCARD B16]** For at least the past six months, to what extent have you been limited because of a health problem in activities people usually do? Would you say that you have been:  过去至少六个月内，您因健康问题导致日常活动受限的程度有多深？您的活动：  Severely limited 严重受限  Limited but not severely 受限，但不严重  Not limited at all 不受限  Not sure/Don’t know 不确定/不知道  Refused 拒绝作答 | Code  1  2  3  99  77 |
| --- | --- | --- |
|  | ***[Interviewer: Proceed to B17 if any of the items on B15a to B15u = 1 (Yes),***  ***Otherwise proceed to Section C]***  ***[采访人员：若B15a到B15u所有项均为1，则进入B17，***  ***否则进入C部分]*** |  |

| B17 | **[SHOWCARD B17] The next questions are about how you manage your health condition(s):**  ***[Interviewer: DK= Don’t Know; NA=Not Applicable]***  **接下来的问题是关于您怎样管理您的健康状况：**  ***[采访人员：DK=不知道；NA=不适用]*** | | | | | | | | | |  | | |
| --- | --- | --- | --- | --- | --- | --- | --- | --- | --- | --- | --- | --- | --- |
|  |  | **Very Little 很少** | |  | **Something**  **知道一些** | | |  | **A Lot**  **了解** | |  | **DK** | **NA** |
| a. | Overall, what I know about my health condition(s) is:  整体上， 我对自己的健康状况的了解程度是: | 0 | 1 | 2 | 3 | 4 | 5 | 6 | 7 | 8 |  | 99 | 88 |
| b. | Overall, what I know about my treatment, including medications of my health condition(s) is：  整体上， 对改善我健康状况的治疗方法， 我了解程度是（包括所使用的药物）: | 0 | 1 | 2 | 3 | 4 | 5 | 6 | 7 | 8 |  | 99 | 88 |
|  |  | **Never**  **从不** | |  | **Sometimes**  **有时** | | |  | **Always**  **每次** | |  | **DK** | **NA** |
| c. | I take medications or carry out the treatments asked by my doctor/health worker  我依从医生或医护人员的指示使用药物或进行医疗 | 0 | 1 | 2 | 3 | 4 | 5 | 6 | 7 | 8 |  | 99 | 88 |
| d. | I share in decisions made about my health condition(s) with my doctor or health worker  我与医生或医护人员按我的健康状况， 一同决定医疗方法 | 0 | 1 | 2 | 3 | 4 | 5 | 6 | 7 | 8 |  | 99 | 88 |
| e. | I am able to deal with health professionals to get the services I need  我能够主动要求医生或医护人员配合我的服务需要 | 0 | 1 | 2 | 3 | 4 | 5 | 6 | 7 | 8 |  | 99 | 88 |
|  |  | **Never**  **从不** | | **Sometimes**  **有时** | | | | | **Always**  **每次** | |  | **DK** | **NA** |
| f. | I attend appointments as asked by my doctor or health worker  我依从医生或医护人员的指示覆诊 | 0 | 1 | 2 | 3 | 4 | 5 | 6 | 7 | 8 |  | 99 | 88 |
| g. | I keep track of my symptoms and early warning signs (e.g. blood sugar levels, peak flow, weight, shortness of breath, pain, sleep problems, mood)  我有监察自己的症状和一些异常警告信号（例如血糖指数、血压 、体重、气喘、疼痛、睡眠状况、情绪等） | 0 | 1 | 2 | 3 | 4 | 5 | 6 | 7 | 8 |  | 99 | 88 |
| h. | I take action when my early warning signs and symptoms get worse  当我身体有异常警告信号和症状转差时， 我有作出处理 | 0 | 1 | 2 | 3 | 4 | 5 | 6 | 7 | 8 |  | 99 | 88 |
|  |  | **Not very well**  **不太好** | |  | **Fairly well**  **一般** | | |  | **Very Well 很好** | |  | **DK** | **NA** |
| i. | I manage the effect of my health condition(s) on my physical activity (i.e. walking, household tasks)  我的活动能力（既是步行， 做家务等）因健康状况受到影响时， 我处理得.. | 0 | 1 | 2 | 3 | 4 | 5 | 6 | 7 | 8 |  | 99 | 88 |
| j. | I manage the effect of my health condition(s) on how I feel (i.e. my emotions and spiritual wellbeing)  我的心情（既是情绪， 心灵健康等）因健康状况受到影响时， 我处理得.. | 0 | 1 | 2 | 3 | 4 | 5 | 6 | 7 | 8 |  | 99 | 88 |
| k. | I manage the effect of my health condition(s) on my social life (i.e. how I mix with other people)  我的社交生活（既是与他人相处——因健康状况受到影响时， 我处理得.. | 0 | 1 | 2 | 3 | 4 | 5 | 6 | 7 | 8 |  | 99 | 88 |
| l. | Overall, I manage to live a healthy life (e.g. no smoking, moderate alcohol, healthy food, regular physical activity, manage stress)  对于过健康生活（例如：不吸烟、不酗酒、健康饮食、恒常运动、管理压力等），整体上我的处理能力是.. | 0 | 1 | 2 | 3 | 4 | 5 | 6 | 7 | 8 |  | 99 | 88 |

| **SECTION C: HEALTH BEHAVIOURS**  **C部分：健康行为** | | | | | | | | |
| --- | --- | --- | --- | --- | --- | --- | --- | --- |
| **The next few questions pertain to personal habits.**  **接下来的问题是关于您的个人习惯。** | | | | | | | | |
| C1a | **[ROUTING QNS]** Have you ever smoked at least 100 cigarettes in your entire life?  您一生中是否抽过至少100支香烟？  Yes 是的  No 没有  Don’t know 不知道  Refused 拒绝作答 | | | | | Code  1  2  99  77 | | Route  C2  C2  C2 |
| C1b | Do you now smoke cigarettes every day, some days, or not at all?  您目前的抽烟状态是？每天，偶尔或是不抽？  Every day 每天  Some days 偶尔  Not at all 不抽  Don’t know 不知道  Refused 拒绝作答 | | | | | Code  1  2  3  99  77 | |  |
| **The next questions will focus on your activities.**  **接下来的问题有关您的活动。** | | | | | | | | |
| C2 | [**SHOWCARD C2]** How often do you do any of the following in the past 6 months?  过去六个月里，您进行下列活动的频率如何？ | | | | | | | |
|  | **Social activities 社交活动** | **Every day**  **每天** | **Every week 每周** | **Every month 每月** | **Less than once a month 一月不到一次** | | **Not at all**  **从不** | |
| a. | Attend Residents’ Committee (RC) / Neighbourhood Committee (NC) / Community Club (CC) / Community Development Council (CDC) / neighbourhood event  参加居委会（RC）/邻里委员会（NC）/社区俱乐部（CC）/社区发展理事会（CDC）/邻里活动 | 1 | 2 | 3 | 4 | | 5 | |
| b. | Attend Senior Activity Centre for activities  参加乐龄中心的活动 | 1 | 2 | 3 | 4 | | 5 | |
| c. | Go out with family members or friends  与家人或朋友出游 | 1 | 2 | 3 | 4 | | 5 | |
| d. | Attends church, mosque or other place of worship  参加教堂、清真寺或其他礼拜活动 | 1 | 2 | 3 | 4 | | 5 | |
|  | **Fitness activities 健身活动** |  |  |  |  | |  | |
| e. | Goes for a walk (for exercise purposes)  散步（为了锻炼运动） | 1 | 2 | 3 | 4 | | 5 | |
| f. | Plays a game of sport / exercise (e.g. Taiji, Qigong, swimming, keep fit or dancing class, etc.)  参加体育/锻炼运动（如太极、气功、游泳、健身或舞蹈班等） | 1 | 2 | 3 | 4 | | 5 | |

| **SECTION D: SOCIAL NETWORK AND SOCIAL ISOLATION**  **D部分: 社交网络和社会隔离** | | | | | | |
| --- | --- | --- | --- | --- | --- | --- |
|  |  | | | | | |
| D1 | **[SHOWCARD D1] The next questions are about how you feel about different aspects of your life. For each one, tell me how often you feel that way....**  **接下来的题是目关于您对人生各方面的想法。在以下每一道题目，试告诉我您多常感觉如此…** | | | | | |
|  |  | **Hardly ever 很少** | **Some of the time**  **有时** | **Often**  **经常** | **Don’t know**  **不知道** | **Refused**  **拒绝作答** |
| a. | How often do you feel that you lack companionship  你多常觉得你缺乏陪伴？ | 1 | 2 | 3 | 99 | 77 |
| b. | How often do you feel left out  您多常觉得自己被冷落？ | 1 | 2 | 3 | 99 | 77 |
| c. | How often do you feel isolated from others  您多常觉得与他人隔绝？ | 1 | 2 | 3 | 99 | 77 |

| D2 | **[SHOWCARD D2] Among all your relatives NOT living with you (including children, grandchildren, in-laws, siblings, nieces, nephews, cousins, uncles, aunties, etc)…**  **在不与您一同生活的所有亲戚中（包括子女、孙子女、姻亲、兄弟姐妹、堂兄弟姐妹、叔叔、阿姨等）** | | | | | | | | |
| --- | --- | --- | --- | --- | --- | --- | --- | --- | --- |
|  |  | **Number of relatives**  **亲戚人数** | | | | | | **Don’t know**  **不知道** | **Refused 拒绝作答** |
| a. | How many relatives do you see or hear from at least once a month?  您有多少位亲戚至少每个月能见他们一面或者和他们通电话？ | 0 | 1 | 2 | 3 - 4 | 5 – 8 | > 9 | 99 | 77 |
| b. | How many relatives do you feel at ease with that you can talk about private matters?  您有多少位亲戚，和他们谈论私人问题时，您感到自在？ | 0 | 1 | 2 | 3 - 4 | 5 - 8 | > 9 | 99 | 77 |
| c. | How many relatives do you feel close to such that you could call on them for help?  您有多少位亲戚已经亲近到可以相互帮助？ | 0 | 1 | 2 | 3 - 4 | 5 - 8 | > 9 | 99 | 77 |

|  |  | | **Never 从不** | | **Seldom 很少** | | **Some**  **Times**  **有时** | | **Often**  **经常** | | **Very Often**  **常常** | **Always 每次** | **Don’t know**  **不知道** | | **Refused 拒绝作答** | |
| --- | --- | --- | --- | --- | --- | --- | --- | --- | --- | --- | --- | --- | --- | --- | --- | --- |
| d. | How often do you see or hear from relatives with whom you have the most contact?  你多常和最多联络次数的亲戚联系？ | | 1 | | 2 | | 3 | | 4 | | 5 | 6 | 99 | | 77 | |
| e. | When one of your relatives has an important decision to make, how often do they talk to you about it?  当你的一位亲戚有重要决定要做时，他们多常向你商量与请教？ | | 1 | | 2 | | 3 | | 4 | | 5 | 6 | 99 | | 77 | |
| f. | How often is one of your relatives available for you to talk to when you have an important decision to make?  当你有重要决定要做时，多常有一位亲戚让你商量与请教？ | | 1 | | 2 | | 3 | | 4 | | 5 | 6 | 99 | | 77 | |
| D3 | **[SHOWCARD D3]** How satisfied are you with your level of contact with relatives?  您对与亲戚之间的联络程度有多满意？  Very satisfied 非常满意  Satisfied 满意  Unsatisfied 不满意  Very unsatisfied 非常不满意  Not sure/Don’t know 不确定/不知道  Refused 拒绝作答 | | | | | | | | | | | | | | | Code  1  2  3  4  99  77 |
| D4 | **[SHOWCARD D4] Among all of your friends including those who live in your neighbourhood…**  **在所有朋友当中（包括您的邻居）** | | | | | | | | | | | | | | | |
|  |  | **Number of friends**  **朋友人数** | | | | | | | | | | | | **Don’t know**  **不知道** | **Refused 拒绝作答** | |
| a. | How many friends do you see or hear from at least once a month?  您有多少位朋友至少每个月能见他们一面或者和他们通电话？ | 0 | | 1 | | 2 | | 3 - 4 | | 5 – 8 | | > 9 | | 99 | 77 | |
| b. | How many friends do you feel at ease with that you can talk about private matters?  您有多少位朋友，和他们谈论私人问题时，您感到自在？ | 0 | | 1 | | 2 | | 3 - 4 | | 5 - 8 | | > 9 | | 99 | 77 | |
| c. | How many friends do you feel close to such that you could call on them for help?  您有多少位朋友已经亲近到可以相互帮助？ | 0 | | 1 | | 2 | | 3 - 4 | | 5 - 8 | | > 9 | | 99 | 77 | |

|  |  | | | | | **Never 从不** | | **Seldom 很少** | | | | | **Some**  **Times 有时** | | | | | **Often 经常** | | **Very Often 常常** | | | **Always 每次** | | | | **Don’t know 不知道** | | | **Refused 拒绝作答** | | | |
| --- | --- | --- | --- | --- | --- | --- | --- | --- | --- | --- | --- | --- | --- | --- | --- | --- | --- | --- | --- | --- | --- | --- | --- | --- | --- | --- | --- | --- | --- | --- | --- | --- | --- |
| d. | How often do you see or hear from friends with whom you have the most contact?  你多常和最多联络次数的朋友联系？ | | | | | 1 | | 2 | | | | | 3 | | | | | 4 | | 5 | | | 6 | | | | 99 | | | 77 | | | |
| e. | When one of your friends has an important decision to make, how often do they talk to you about it?  当你的一位朋友有重要决定要做时，他们多常向你商量与请教？ | | | | | 1 | | 2 | | | | | 3 | | | | | 4 | | 5 | | | 6 | | | | 99 | | | 77 | | | |
| f. | How often is one of your friends available for you to talk to when you have an important decision to make?  当你有重要决定要做时，多常有一位朋友让你商量与请教？ | | | | | 1 | | 2 | | | | | 3 | | | | | 4 | | 5 | | | 6 | | | | 99 | | | 77 | | | |
| D5 | **[SHOWCARD D5]** How satisfied are you with your level of contact with friends?  您对与朋友之间的联络程度有多满意？  Very satisfied 非常满意  Satisfied 满意  Unsatisfied 不满意  Very unsatisfied 非常不满意  Not sure/Don’t know 不确定/不知道  Refused 拒绝作答 | | | | | | | | | | | | | | | | | | | | | | | | | | | | | | | Code  1  2  3  4  99  77 | |
| D6 | **[SHOWCARD D6] Among all of your neighbours including those you consider your friend…**  **在所有邻居当中，包括作为朋友的邻居。。。** | | | | | | | | | | | | | | | | | | | | | | | | | | | | | | | | |
|  |  | | | | | | **Number of neighbours**  **邻居人数** | | | | | | | | | | | | | | | | | | | | | **Don’t know**  **不知道** | | | **Refused 拒绝作答** | | |
| a. | How many neighbours do you see or hear from at least once a month?  您有多少位邻居至少每个月能见他们一面或者和他们通电话？ | | | | | | 0 | | 1 | | | | | 2 | | | | | 3 - 4 | | 5 – 8 | | | > 9 | | | | 99 | | | 77 | | |
| b. | How many neighbours could you call on for help?  您有多少位邻居可以请求帮助？ | | | | | | 0 | | 1 | | | | | 2 | | | | | 3 - 4 | | 5 - 8 | | | > 9 | | | | 99 | | | 77 | | |
|  | |  |  |  | | | | | | | |  | | | |  | | | | | | | | |  | | | | | | | | |
| **SECTION E: PSYCHOLOGICAL STATE AND STRESSORS**  **E部分：精神状态和压力因素** | | | | | | | | | | | | | | | | | | | | | | | | | | | | | | | | | |
| E1 | **[SHOWCARD E1] The two following questions are on your psychological state. These questions commonly used in surveys, and it is important that we receive your answers for comparative purposes. We ask that you bear with us and thank you for your cooperation.**  **接下来的两个问题关于您的精神状态。这些问题通常用于调查，我们需要获得您的答案用于比较之用，这点尤为重要。请多多包涵，给予我们一些耐心，谢谢您的配合。** | | | | | | | | | | | | | | | | | | | | | | | | | | | | | | | | |
|  | **Over the past two weeks, how often have you been bothered by any of the following problems?** *[Interviewer: Should respondent have any query, you may explain as such: There may be some questions for which you have no answer or which seem the same as another question, but the same questions are used internationally in studies and tests. We ask for your full cooperation.]*  **在过去的两周里，您感觉自己被以下症状所困扰的频率是？**  *[采访人员：若回答者有任何疑问，你需要照此解释：可能会有一些问题您不能回答或看似与其他问题一样，但这些相同的问题是研究测市国际通用问题。希望您能完全配合。]* | | | | | | | | | | | | | | | | | | | | | | | | | | | | | | | | |
|  |  | | | | | | **Not at all**  **完全没有** | | | | **Several days**  **有过几天-**  **（少过一周）** | | | | | | **More than half the days**  **超过一半天数**  **（一周）** | | | | | **Nearly every day**  **几乎每天** | | | | **Don’t know 不知道** | | | **Refused 拒绝作答** | | | | |
| a. | Little interest or pleasure in doing things  对任何事情都提不起兴趣  /感受不到兴趣 | | | | | | 0 | | | | 1 | | | | | | 2 | | | | | 3 | | | | 99 | | | 77 | | | | |
| b. | Feeling down, depressed, or hopeless  感觉沮丧的，忧郁的，或绝望的 | | | | | | 0 | | | | 1 | | | | | | 2 | | | | | 4 | | | | 99 | | | 77 | | | | |
| E2 | **[SHOWCARD E2] The following list of events is extremely stressful and upsetting events that sometimes occur to people. Can you indicate which, if any, of these events you have experienced in your life, over the past year? [MA]**  **以下列表列出了种种可能让人承受极度压力或不安的事件。如果您在过去的一年里有过同样的经历，请您指出。 [MA]** | | | | | | | | | | | | | | | | | | | | | | | | | | | | | | | | |
|  | **Events 事件** | | | | | | | | | | | | | | | | | | | | | | | | | | | | | | | **Code** | |
| a. | Spouse/partner died  配偶/伴侣过世 | | | | | | | | | | | | | | | | | | | | | | | | | | | | | | | 1 | |
| b. | Spouse/partner have life threatening illness or accident  配偶/伴侣经历危及生命的疾病或意外 | | | | | | | | | | | | | | | | | | | | | | | | | | | | | | | 2 | |
| c. | A family or close friend died/ have life threatening illness or accident (other than your spouse or partner)  家人或亲密朋友死亡/经历危及生命的疾病或意外（除了您的配偶或伴侣） | | | | | | | | | | | | | | | | | | | | | | | | | | | | | | | 3 | |
| d. | Had major problems with money 经济上有重大问题 | | | | | | | | | | | | | | | | | | | | | | | | | | | | | | | 4 | |
| e. | Divorced or breakup 离婚或分手 | | | | | | | | | | | | | | | | | | | | | | | | | | | | | | | 5 | |
| f. | Major conflict with children or grandchildren  与孩子或孙子女有重大的冲突 | | | | | | | | | | | | | | | | | | | | | | | | | | | | | | | 6 | |
| g. | Major accidents/disasters/muggings/unwanted sexual experiences/robberies  遭遇重大事故/灾难/殴打/强迫性行为/抢劫 | | | | | | | | | | | | | | | | | | | | | | | | | | | | | | | 7 | |
| h. | Physical abused or threatened  遭肉体上的虐待或威胁 | | | | | | | | | | | | | | | | | | | | | | | | | | | | | | | 8 | |
| i. | Verbally abused  遭言语虐待 | | | | | | | | | | | | | | | | | | | | | | | | | | | | | | | 9 | |
| j. | Pet died  宠物死亡 | | | | | | | | | | | | | | | | | | | | | | | | | | | | | | | 10 | |
| k. | Family member/Close friend lost job/retire  家庭成员/亲密朋友失去工作/退休 | | | | | | | | | | | | | | | | | | | | | | | | | | | | | | | 11 | |
| l. | None of the above以上从未发生 | | | | | | | | | | | | | | | | | | | | | | | | | | | | | | | 12 | |
| m. | Refused拒绝作答 | | | | | | | | | | | | | | | | | | | | | | | | | | | | | | | 77 | |
| **SECTION F: INCOME**  **F部分：收入** | | | | | | | | | | | | | | | | | | | | | | | | | | | | | | | | | |
| F1 | **[SHOWCARD F1] What is your largest source of funds? Please select from the following the 3 main sources indicating their order of importance.**  **您主要的资金来源是什么？请从以下选择，将3个主要来源按重要性顺序排列。** | | | | | | | | | | | | | | | | | | | | | | | | | | | | | | | | |
|  | First source 第一个来源 | | | |  | | | | | | | | | | | | | | | | | | | | | | | | | | | | |
|  | Second source 第二个来源 | | | |  | | | | | | | | | | | | | | | | | | | | | | | | | | | | |
|  | Third source 第三个来源 | | | |  | | | | | | | | | | | | | | | | | | | | | | | | | | | | |
|  |  | | | | | | | | | | | | | | | | | | | | | | | | | | | | | | | | |
| **[SHOWCARD F1]** | | | | | | | | | | | | | | | | | | | | | | | | | | | | | | | | | |
| 1 | Income from work 工作收入 | | | | | | | | | 5 | | | | | Financial support from children, grandchildren or relatives 子孙或亲属财务支持 | | | | | | | | | | | | | | | | | | |
| 2 | Pension 养老金 | | | | | | | | | 6 | | | | | Public assistance / Short-term financial assistance from Community Development Councils (CDC) 公共援助/社区发展理事会（CDC）短期经济援助 | | | | | | | | | | | | | | | | | | |
| 3 | Central Provident Fund 公积金 | | | | | | | | | 7 | | | | | Other (Please specify: ________ ) 其他（请注明：____________________________） | | | | | | | | | | | | | | | | | | |
| 4 | Savings, life insurance, bonds, stock 存款、人寿保险、债券、股份 | | | | | | | | | 8 | | | | | Not sure 不确定 | | | | | | | | | | | | | | | | | | |
|  |  | | | | | | | | | | | | | | | | | | | | | | | | | | | | | | | | |
| F2 | **[SHOWCARD F2] Do you feel that you have adequate funds to meet your expenses per month?**  **您认为以您每个月所获得的收入，是否足够维持每个月的花销？**  Enough money, with some left over 够用，有剩余的储蓄  Just enough money, no difficulty 刚好够用，没大碍  Some difficulty to meet expenses 满足花销有些难度  Much difficulty to meet expenses 满足花销非常困难  Not sure/Don’t know 不确定/不知道  Refused 拒绝作答 | | | | | | | | | | | | | | | | | | | | | | | | | | | | | | | | Code  1  2  3  4  99  77 |

| **SECTION G: HEALTH CARE UTILIZATION**  **G部分：医疗服务利用率** | | | | |  | |
| --- | --- | --- | --- | --- | --- | --- |
| G1 | **Now I would like to ask you about your contact with health professionals and services**  **以下问题是关于您与健康专业人士和服务机构的联系** | | | | | |
| **(i) During the last six months, did you (subject):**  **(i) 过去六个月内，您是否：** | | **No**  **没有** | **Yes**  **是的** | **(ii) Number of times in the last six months**  **（ii）过去六个月内次数** | | **(iii) Number of different doctors or practitioners seen in the last six months**  **（iii）过去六个月内向不同的医生或执业医师就诊次数** |
|  | see a GP in a private clinic?  在私人诊所就诊全科医生？ | 2  **[Go to b]** | 1 🡪 | Times/次 | |  |
|  | see a doctor in a polyclinic?  就诊于综合诊疗所？ | 2  **[Go to c]** | 1 🡪 | Times/次 | |  |
|  | see a doctor in a specialist outpatient clinic?  就诊于专科门诊？ | 2  **[Go to d]** | 1 🡪 | Times/次 | |  |
|  | see a doctor at home?  在家看病？ | 2  **[Go to e]** | 1 🡪 | Times/次 | |  |
|  | see a doctor at a senior activity center or at any volunteer organizations  在老年人活动中心或任何志愿机构看医生 | 2  **[Go to f]** | 1 🡪 | Times/次 | |  |
|  | see a Traditional Chinese Medicine (TCM) practitioner or a traditional healer?  就诊中医（TCM）执业医生或传统治疗师？ | 2 | 1 🡪 | Times/次 | |  |
| Total | | | | | |  |
|  | | | | | |  |

| ***[Interviewer proceed to G2 if:***   - ***Sum of G1(iii) a-e >1***   ***Otherwise, proceed to G4a]*** | | | |
| --- | --- | --- | --- |
| G2 | **[ROUTING QNS] You report that you saw at least two different doctors or practitioners in the last six months. Were these consultations for the same health problem you were facing?**  ***[采访人员：开始下一项前请阅读上述说明]***  **您说过去六个月内您至少和两名医生或执业医师就诊。每次就诊均是针对您面对的同一健康问题吗？**  Yes 是的  No 没有  Not sure/Don’t know 不确定/不知道  Refused 拒绝作答 | Code  1  2  99  77 | Route  G4a  G4a |
| G3 | **[SHOWCARD G3] How often were you confused about the advice provided by the different doctors or practitioners?**  **您多常因不同医生或执业医师提供的建议而感到困惑？**  Never 从不  Rarely 很少  Sometimes 有时  Frequently 多时  Always 常常  Not sure/Don’t know 不确定/不知道  Refused 拒绝作答 | Code  1  2  3  4  5  99  77 |  |

| G4a | **[ROUTING QNS]** In the past six months, have you been to a hospital for medical treatment, exams or test?  过去六个月内，您有去医院接受治疗、检查或检测吗？  Yes 有  No 没有  Not sure/Don’t know 不确定/不知道  Refused 拒绝作答 | | | | | | Code  1  2  99  77 | Route  G4b  G5  G5  G5 |
| --- | --- | --- | --- | --- | --- | --- | --- | --- |
| G4b | In the past **six months**, which hospitals have you been to for medical treatment, exams, or tests?  过去六个月内，您在哪些医院接受了治疗、检查或检测？ | 1. | | | 4. | | | |
|  |  | 2. | | | 5. | | | |
|  |  | 3. | | | 6. | | | |
| G5 | **Now, we would like to know if you are receiving any supports near your neighbourhood.**  **Please tell us if you are accessing any of the following services in the last 6 months:**  **[NOTE to Interviewers: All 3 options (i.e. “Yes”, “No” and “Never heard of it”) should be read out to the subject]**  **现在，我们想知道您是否收到了附近邻里的援助。**  **您在过去6个月内是否接受过以下服务：** | | | | | | | |
|  |  | | **YES 是的** | **NO 没有** | | **Don’t know/ Never heard of it 不知道/从未听说** | | |
| a. | Day care centers (including senior activity centers, dementia day care centers, or day rehab centers)  日托中心（包括老年人活动中心、痴呆患者日托中心或日常康复中心） | | 1 | 2 | | 3 | | |
| b. | Home health services e.g. nurse, doctor, therapist etc.  家庭护理服务，如护士、医生理疗师等 | | 1 | 2 | | 3 | | |
| c. | Home help services e.g. for cleaning, delivering meals, etc.  家政服务，如清洁、送饭等 | | 1 | 2 | | 3 | | |
| d. | Caregiver support services or respite care  护工支持服务或临时看护 | | 1 | 2 | | 3 | | |
| e. | Home modifications e.g. installation of grab bars, ramps, anti-slip mats  房屋装修，如安装扶手杆、坡道、防滑垫 | | 1 | 2 | | 3 | | |
| f. | Visits from “Befrienders” or other volunteers for social interactions  “益友”或其他志愿者社会互动项目拜访 | | 1 | 2 | | 3 | | |
| g. | Escort services and transportation services  陪护服务和交通服务 | | 1 | 2 | | 3 | | |
| h. | Any other, please specify: _______________________________  其他，请注明：____________________________ | | 1 | 2 | | 3 | | |

| **SECTION H: MEDICATION ADHERENCE**  **H部分：药物依从性** | | | | |  | |  |
| --- | --- | --- | --- | --- | --- | --- | --- |
| H1 | **The next set of questions is specifically about the medications you take.**  **接下来的问题特别关于您的服药情况。** | | | | | | |
|  | | **Yes 是的** | **No 没有** | **Don’t know 不知道** | | **Refused**  **拒绝作答** | |
| a. | Are you taking any prescription medications?  您在服用任何处方药吗？ | 1 | 2 [Go to I1] | 99 | | 77 | |
| b. | Do you sometimes forget to take your pills?  您是否有时会忘记服药？ | 1 | 2 | 99 | | 77 | |
| c. | People sometimes miss taking their medications for reasons other than forgetting. Thinking over the past two weeks, were there any days when you did not take your medicine?  人们偶尔会错过服药，并非因为忘记。回想过去两周，您是否有几天未服药？ | 1 | 2 | 99 | | 77 | |
| d. | Have you ever cut back or stopped taking your medicine without telling your doctor because you felt worse when you took it?  您是否减少或停止服用药物而没有告知其医生，因为您感觉服药后情况更糟？ | 1 | 2 | 99 | | 77 | |
| e. | When you feel like your symptoms are under control, do you sometimes stop taking your medicine?  当您感觉症状得到控制后，是否偶尔停药？ | 1 | 2 | 99 | | 77 | |
| H2 | **How many different prescription medications do you take on a regular basis?**  您经常服用几种不同处方药？  _______________________ / Don’t know不知道/ Not sure不确定 | | | | | | |

| **SECTION I: QUALITY OF LIFE**  **I部分：生活质量** | | |
| --- | --- | --- |
| **I1** | **[SHOWCARD I1]** For the following aspects, please select the ONE statement that best describes your health TODAY.  在以下各方面，请选择最适当的句子来形容您目前的状况。 | |
| a | MOBILITY 行动方面 |  |
|  | I have no problems in walking about 我走动没有问题。 | 1 |
|  | I have slight problems in walking about 我走有轻微问题。 | 2 |
|  | I have moderate problems in walking about 我走动有中度问题。 | 3 |
|  | I have severe problems in walking about 我走动方面有严重问题。 | 4 |
|  | I am unable to walk about 我无法到处走动。 | 5 |
| **b** | SELF-CARE 自顾方面 |  |
|  | I have no problems washing or dressing myself 我梳洗和穿衣服没有问题。 | 1 |
|  | I have slight problems washing or dressing myself 我梳洗和穿衣服有轻微问题。 | 2 |
|  | I have moderate problems washing or dressing myself 我梳洗和穿衣服有中度问题。 | 3 |
|  | I have severe problems washing or dressing myself 我梳洗和穿衣服有严重问题。 | 4 |
|  | I am unable to wash or dress myself 我无法梳洗我自己，也不能自己穿衣服。 | 5 |
| **c** | **USUAL ACTIVITIES** *(e.g. work, study, housework, family or leisure activities)* **日常活动***(例如，工作，温书，做家务，家庭或消闲活动)* | |
|  | I have no problems doing my usual activities 我日常活动没有问题。 | 1 |
|  | I have slight problems doing my usual activities 我日常活动有轻微问题。 | 2 |
|  | I have moderate problems doing my usual activities 我日常活动有中度问题。 | 3 |
|  | I have severe problems doing my usual activities 我日常活动有严重问题。 | 4 |
|  | I am unable to do my usual activities 我无法做日常活动。 | 5 |
| **d** | **PAIN / DISCOMFORT疼痛/不适** |  |
|  | I have no pain or discomfort 我没有感觉疼痛或者不适。 | 1 |
|  | I have slight pain or discomfort 我有感觉轻微的疼痛或者不适。 | 2 |
|  | I have moderate pain or discomfort 我有感觉中等的疼痛或者不适。 | 3 |
|  | I have severe pain or discomfort 我有感觉严重的疼痛或者不适。 | 4 |
|  | I have extreme pain or discomfort 我感觉极端的疼痛或者不适。 | 5 |
| **e** | **ANXIETY / DEPRESSION 焦虑 / 抑郁** |  |
|  | I am not anxious or depressed 我不焦虑或抑郁。 | 1 |
|  | I am slightly anxious or depressed 我有点焦虑或抑郁。 | 2 |
|  | I am moderately anxious or depressed 我有中等的焦虑或抑郁。 | 3 |
|  | I am severely anxious or depressed 我有严重的焦虑或抑郁。 | 4 |
|  | I am extremely anxious or depressed 我有极端的焦虑或抑郁。 | 5 |

| I2 | [SHOWCARD I2] Interviewer: Instruct subject to use scale on the right hand side of this page to answer this question.  [SHOWCARD I2] 访员：让受访者使用本页右侧的刻度，回答这个问题。  Please rate how good or bad your health TODAY, on a scale from 0 to 100. A score of 100 means the best of health you can imagine, while a score of 0 means the worst health you can imagine. [SA]  请从0到100的分数，评价您今天的健康状况的好坏。100分的指数表示您最理想的健康状况，而0分则表示您想象中最糟的健康状况。[SA] |
| --- | --- |


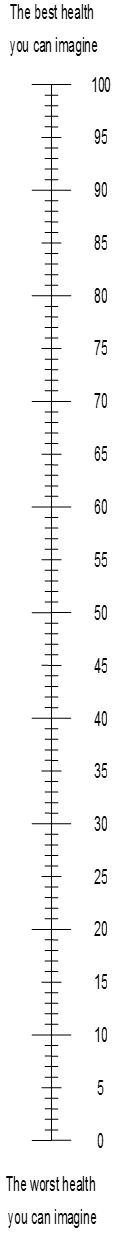


|  |
| --- |
