# Supplementary material for: Do healthcare needs-based population segments predict outcomes among the elderly? Findings from a prospective cohort study in an urbanized low-income community
Source: BMC Geriatr. 2020 Feb 27;20:78. doi: 10.1186/s12877-020-1480-9 (PMC7045405; doi:10.1186/s12877-020-1480-9)
Supplement: Supplementary file 5 — Additional file 5. Comparison of actionability. Comparison of all 4 segmentation schemes in this study in terms of number and type of healthcare need type variables utilized as input to the respective schemes. [file 12877_2020_1480_MOESM5_ESM.docx]

| **Healthcare need type variable utilized in segmentation scheme** | **Singapore** | **Lombardy** | **Delaware** | **North-West London** |
| --- | --- | --- | --- | --- |
| Medical condition | Yes | Yes | Yes | Yes |
| Geriatric syndrome  (E.g. recurrent falls/gait instability, frailty, etc.) | Yes | No | No | No |
| Social support | Yes | No | No | Yes |
| Psychiatric and behavioural issues  (E.g. confusion/ forgetfulness, depression, anxiety, etc.) | Yes | No | Yes | Yes |
| Physical function  (E.g. activity of daily living independence, etc.) | Yes | No | No | Yes |
| Skilled task needs  (E.g. nursing and rehabilitative tasks) | Yes | No | No | No |
| Patient activation | Yes | No | No | No |
| Recent healthcare utilization | Yes | Yes | No | No |
| Polypharmacy | No | Yes | No | No |
| Maternal and child health | No | Yes | Yes | No |
| Total ‘yes’ | 8 | 4 | 3 | 4 |

Comparison of actionability between the Singapore, Lombardy, Delaware and North-West London population segmentation schemes

When a segmentation scheme segments a population based on a higher variety of healthcare need type variables, the resulting population segments provides more granular insights to policy makers regarding what kind of health services would likely meet needs within a segment as well as the means of service provisions which are suitable given the characteristic of segment subjects (1, 2). By extension, subjects within homogenous segments would also tend to be more similar in terms of healthcare priorities and utilization patterns (3). In addition, healthcare need type variables include patient characteristics such as ‘Patient Activation’ which aids decisions on selection of appropriate services by excluding potentially needed services that are contraindicated for poorly activated individuals.

**References**

1. Chong JL, Matchar DB. Benefits of Population Segmentation Analysis for Developing Health Policy to Promote Patient-Centred Care. Ann Acad Med Singapore. 2017;46(7):287-9.

2. Vuik SI, Mayer EK, Darzi A. Patient Segmentation Analysis Offers Significant Benefits For Integrated Care And Support. Health Affairs. 2016;35(5):765-79.

3. Lynn J, Straube BM, Bell KM, Jencks SF, Kambic RT. Using population segmentation to provide better health care for all: the "Bridges to Health" model. Milbank Quarterly. 2007;85(2).
